# IL-4 Causes Hyperpermeability of Vascular Endothelial Cells through Wnt5A Signaling

**DOI:** 10.1371/journal.pone.0156002

**Published:** 2016-05-23

**Authors:** Tom Skaria, Julia Burgener, Esther Bachli, Gabriele Schoedon

**Affiliations:** 1 Inflammation Research Unit, Department of Medicine, Division of Internal Medicine, University Hospital Zürich, Zürich, Switzerland; 2 Department of Medicine, Uster Hospital, Uster, Switzerland; Texas A&M University Health Science Center College of Medicine & Baylor Scott and White Health, UNITED STATES

## Abstract

Microvascular leakage due to endothelial barrier dysfunction is a prominent feature of T helper 2 (Th2) cytokine mediated allergic inflammation. Interleukin-4 (IL-4) is a potent Th2 cytokine, known to impair the barrier function of endothelial cells. However, the effectors mediating IL-4 induced cytoskeleton remodeling and consequent endothelial barrier dysfunction remain poorly defined. Here we have used whole genome transcriptome profiling and gene ontology analyses to identify the genes and processes regulated by IL-4 signaling in human coronary artery endothelial cells (HCAEC). The study revealed Wnt5A as an effector that can mediate actin cytoskeleton remodeling in IL-4 activated HCAEC through the regulation of LIM kinase (LIMK) and Cofilin (CFL). Following IL-4 treatment, LIMK and CFL were phosphorylated, thereby indicating the possibility of actin stress fiber formation. Imaging of actin showed the formation of stress fibers in IL-4 treated live HCAEC. Stress fiber formation was notably decreased in the presence of Wnt inhibitory factor 1 (WIF1). Non-invasive impedance measurements demonstrated that IL-4 increased the permeability and impaired the barrier function of HCAEC monolayers. Silencing Wnt5A significantly reduced permeability and improved the barrier function of HCAEC monolayers upon IL-4 treatment. Our study identifies Wnt5A as a novel marker of IL-4 activated vascular endothelium and demonstrates a critical role for Wnt5A in mediating IL-4 induced endothelial barrier dysfunction. Wnt5A could be a potential therapeutic target for reducing microvascular leakage and edema formation in Th2 driven inflammatory diseases.

## Introduction

Interleukin-4 (IL-4) is a multifunctional pleiotropic type I cytokine secreted by activated T helper (Th) 2 cells, basophils, eosinophils and mast cells [1, 2]. Functionally, IL-4 induces the differentiation of antigen stimulated naïve T cells to a Th2 phenotype [3, 4]. It also regulates immunoglobulin (Ig) class switching so that B lymphocytes express IgE [5], and regulates apoptosis, cell proliferation and expression of several genes in different cell types such as fibroblasts, macrophages, endothelial and epithelial cells [1, 2]. IL-4 drives the ‘alternative activation of macrophages’ to produce the M2 phenotype, which is crucially involved in type 2 immunity. Thus, through its effects on multiple cell types and by binding to alternative cell surface receptors [1], IL-4 plays critical roles in allergic inflammation [6], immune response to extracellular parasites including helminths, autoimmunity [2] and tumor inflammation and metastasis [1, 7].

Recent evidence suggests a potential role for IL-4 in generating a proinflammatory environment in vascular endothelial cells (VEC) [8]. VEC are essential for maintaining vascular homeostasis in normal physiological conditions [9]. Under pathophysiological conditions, activated blood components, pathogens or inflammatory mediators such as cytokines act upon VEC and heavily alter their functions, conferring on them an inflamed phenotype. These alterations include a change from the anticoagulant phenotype to a procoagulant state, increased production of vasoactive substances, expression of cell adhesion molecules, synthesis of inflammatory mediators including chemoattractants, and endothelial barrier dysfunction causing microvascular leakage. It has been demonstrated that IL-4 upregulates the expression of vascular cell adhesion molecule (VCAM)-1 [10–12], IL-6 [12–14] and monocyte chemotactic protein (MCP) [12, 14] in human umbilical vein endothelial cells (HUVEC). Moreover, stimulation with IL-4 increased the adhesion of peripheral blood monocytes [15] and T cells [16] to HUVEC. IL-4 has been shown to induce cytoskeletal rearrangements in HUVEC and significantly regulate their proliferation [17]. Further, IL-4 acts as a modest mitogen for both macro and microvascular endothelial cells [8, 17–19]. IL-4 has proatherogenic effects and induces the apoptosis of endothelial cells causing increased endothelial cell turnover [20]. It has been demonstrated that IL-4 induces hyperpermeability of HUVEC, causing vascular leakage [21], however, effectors responsible for IL-4 induced endothelial hyperpermeability and consequent barrier dysfunction remain unidentified. Moreover, these previous studies addressing the effects of IL-4 on endothelial inflammation used HUVEC as the primary endothelial cell model system. HUVEC that are obtained from the immune naïve foetal tissue shows significant variations in function compared with adult vascular endothelium and hence may be an inappropriate primary cell model of vascular endothelium [22]. The targets and effects of IL-4 signaling in adult VEC, therefore, may vary from HUVEC and have to be elucidated.

In the present study, we used transcriptome profiling to identify the genes regulated by paracrine IL-4 signaling in our established *in vitro* model of adult VEC, cultured human coronary artery endothelial cells (HCAEC) [23]. Here we identify Wnt5A as one of the genes significantly upregulated by IL-4 treatment. We further demonstrate a critical role for IL-4 induced Wnt5A in impairing barrier function of endothelial monolayers. Our findings suggest a prominent role for Wnt5A in causing microvascular leakage associated with IL-4 driven allergic inflammation and other pathophysiological conditions.

## Materials and Methods

### Cell Culture

HCAEC and human pulmonary artery endothelial cells (HPAEC) purchased from Clonetics (Lonza, USA) were cultured in EBM-2 medium (Clonetics, Lonza, USA) supplemented with EGM-2MV Single Quots and 5% FBS (Clonetics, Lonza) as described previously [23]. For experiments, cells at passages three to six were used and the serum in culture medium was decreased to 2% FBS. Macrophages derived from human PBMC were cultured as described [24]. Cells were cultured under standard conditions (37°C, 5% CO_2_, 80% humidity) in a Class 100 HEPA air filtered system (SteriCult, Fisher Scientific, Switzerland). Culture medium without antibiotics was used to prevent masked low-level contamination in cell cultures. Treatments were carried out using recombinant human (rh) IL-4 (4 ng/mL; purity >98%; PeproTech, USA), rh IL-6 (20 U/mL; purity >98%; PeproTech, USA), rh soluble Frizzled-related peptide-1 (sFRP1; 10 μg/mL; purity > 95%; R&D systems, USA), rh/mouse Wnt5A (250 ng/mL; CHO-derived Gln38-Lys380, purity >80%, endotoxin level <1.0 EU/μg of protein; R&D systems, USA) and rh Wnt inhibitory factor 1 (WIF1; 15 μg/mL; purity >97%; R&D systems, USA). Sterile biopure ep Dualfilter T.I.P.S. sterile filter tips (Eppendorf, Germany) were used throughout the study.

### RNA Isolation and Quantitative Real-Time PCR (qRT-PCR) Analyses

RNA isolation and qRT-PCR using the HPRT gene as endogenous control were performed as described previously [25]. Sequence specific PCR detection primers used for Wnt5A, IL-6 and HPRT are as follows: Wnt5A forward, 5'-AGT TGC CTA CCC TAG C-3'; Wnt5A reverse, 5'-GTG CCT TCG TGC CTA T-3'; IL-6 forward, 5'-CCT GAC CCA ACC ACA AA-3'; IL-6 reverse, 5'-AGT GTC CTA ACG CTC ATA C-3'; HPRT forward, 5'-CCA GTC AAC AGG GGA CAT AAA-3'; HPRT reverse, 5'-CAC AAT CAA GAC ATT CTT TCC AGT-3'. Thermal cycling conditions set in the 7500 Fast Real-Time PCR System (Applied Biosystems, USA) involved an initial denaturation step (10 min, 95°C) followed by 40 cycles of denaturation (15 sec, 95°C), annealing (30 sec, 55°C), and extension (30 sec, 72°C).

### Differential Gene Expression Profiling

Microarray based gene expression profiling and scanning, feature extraction and data normalization of microarrays were performed as described previously [25]. Complete data sets for the IL-4 regulated transcriptome in HCAEC are available in the NCBI GEO data repository, accession number GSE64860.

### Microarray Data Analyses

The preprocessed microarray data was analyzed using GeneSpring GX 9.0 Software (Agilent Tech. Inc.) with default settings for two color arrays and a fold change (FC) cutoff of 2. Using MetaCore™ GeneGO software (Thomson Reuters, http://portal.genego.com), genes regulated ≥2 fold in their expression and satisfying a *P* value <0.05 were grouped into pathways according to their biologic functions and gene ontology (GO) classes.

### Immunofluorescence Staining

HCAEC were grown in four-chamber culture slides coated with rat tail collagen type I (diluted 0.01% in sterile pyrogen-free water; BD Biosciences, USA). To stain for VE-cadherin, HCAEC were grown to a confluent monolayer. After treatment with appropriate stimuli, cells were fixed with 4% formalin in PBS for 20 min. After washing three times with PBS (pH 7.4) at room temperature (RT), slides were treated with blocking solution for 1 h at RT. Depending on the source of secondary antibodies, blocking solution containing either 10% goat serum and 1% BSA (for secondary antibodies derived from goat) or 10% rabbit serum and 1% BSA in PBS (for secondary antibodies derived from rabbit) was used. After washing three times with PBS at RT, slides were incubated with primary antibodies diluted in blocking solution at 4°C overnight. The following antibodies were used with dilutions indicated: rabbit anti-VE-cadherin, polyclonal (1:100; Product No. 2158, Cell Signaling Technology), rabbit anti-phospho LIMK2, polyclonal (pT505; 1:300; Product No. Ab131343, Abcam), rabbit anti-phospho CFL, polyclonal (pS3; 1:500; Product No. Ab100836, Abcam), goat anti-Wnt5A, polyclonal (1:500; Cat. No. AF645, R&D Systems). Slides were then washed three times with PBS at RT. Depending on the first antibody’s species, slides were incubated with Alexa 568 labelled goat anti-rabbit or rabbit anti-goat secondary antibodies (1:2000; Molecular Probes, Invitrogen, USA) for 1 h at RT in the dark. Incubations with secondary antibody were combined with Alexa Fluor 488 Phalloidin (1:80; Molecular Probes, Invitrogen, USA) to visualize F-actin. Slides were washed three times with PBS at RT, counterstained with 10 μg/mL diamidino-phenylindole (DAPI; Sigma-Aldrich) and mounted using ProLong® Gold Antifade (Life Technologies, USA). Images were captured using an Axioskope microscope equipped with an AxioCam MRm digital camera and the associated AxioVision Rel.4.6 software (Carl Zeiss, Feldbach Switzerland). Cellular fluorescent intensity was quantified from five different areas of the respective fluorescent images using ImageJ based Fiji software (Fiji is just ImageJ) and corrected for background fluorescence.

### Immunoblotting

Immunoblotting was performed as described previously with some modifications [26]. Equal amounts of protein were resolved on Criterion Stain-Free Precast Gels (Cat. 567–8074, Bio-Rad) and transferred onto PVDF membranes. After incubating with blocking solution (5% BSA in TBS containing 0.1% Tween-20), membranes were probed with primary antibodies diluted in blocking solution at 4°C overnight. The following antibodies were used with dilutions indicated: rabbit anti-phospho LIMK2, polyclonal (1:500; Product No. Ab131341, Abcam), rabbit anti-LIMK2, polyclonal (1:1000; Product No. PA5-36062, ThermoFischer Scientific), rabbit anti-phospho CFL, polyclonal (1:1000; Product No. Ab100836, Abcam), rabbit anti- CFL, polyclonal (1:1000; Product No. Ab42824, Abcam), goat anti-Wnt5A, polyclonal (1:200; Cat. No. AF645, R&D Systems). Blots were then washed, incubated with anti-rabbit IgG- HRP-linked whole antibody (1:5000; Cat. No. NA934V, GE Healthcare UK Limited) or anti-goat IgG-HRP (1:2000; Cat. No. P0160, Dako Cytomation) for 1 h at RT and visualized with ECL Plus Western blotting detection reagents (Amersham Bioscience) in a ChemiDoc MP imaging system (Bio-Rad). Blots were analyzed by densitometry using Bio-Rad Image lab 5.2.1 software. For quantification of phosphoproteins, ratios of pLIMK2/LIMK2, and pCFL/CFL were calculated. In-gel stained total protein served as loading control for Wnt5A protein quantification. Data are expressed as mean ± SEM from three independent experiments.

### Inhibition of Rho associated protein serine/threonine kinases (ROCK)

Y-27632 (Calbiochem; Millipore, USA) at a concentration of 10 μM [27] was used to inhibit the activation of ROCK. ROCK inhibition experiments were performed by incubating cells in medium containing Y-27632 alone or in combination with IL-4 for 1 h and 4 h.

### Live Cell Imaging of Actin

HCAEC monolayers were grown in rat tail collagen type I coated optical 96 microwell culture plates (Thermo Scientific, USA). Cells were incubated with CellLight® Actin-RFP probe (Cat. No. C10583, Life Technologies, USA) for 16 h (time needed for probe transfection/incorporation) according to manufacturer’s instructions. After medium change, cells in triplicate wells were treated with IL-4 or Wnt5A alone or in combination with sFRP1 or WIF1. Cells were further incubated up to 24 h. At the time indicated, images of live actin cytoskeleton organization were obtained using the Axio Observer.Z1 inverted microscope equipped with an AxioCam MRm digital camera and the associated ZEN 2012 software (Carl Zeiss, Feldbach, Switzerland).

### Wnt5A Silencing

To knockdown Wnt5A expression, HCAEC grown up to 80% confluency in 24-well plates were incubated with transfection complexes formed from Wnt5A-siRNA (5 nM; Hs_Wnt5A_6, Cat. No. SI03025596; Qiagen) and 6 μL/mL HiPerFect Transfection reagent (Qiagen GmbH, Hilden, Germany) in EBM-2 basal medium (Clonetics, Lonza, USA) for 24 h. After splitting using Trypsin EDTA, transfected cells were seeded onto collagen coated 6 well plates, retransfected for an additional round as above and incubated until the cells reached 90% confluency. HCAEC were also transfected with validated AllStars negative control siRNA (Qiagen) in parallel to control for ‘off target’ effects of siRNAs. Cells were then either treated with IL-4 for 8 h and lysed (for qRT-PCR analyses) or trypsinized and seeded into collagen-coated 8W10E+ arrays (for barrier function assays, see below). Treatments were carried out in EGM-2 MV medium with 2% FBS devoid of transfection complexes. To immunostain for Wnt5A, HCAEC transfected once with Wnt5A-siRNA were harvested by trypsinization and seeded into collagen-coated BD Falcon 4 chamber culture slides (BD Biosciences, USA). There, an additional round of Wnt5A-siRNA transfection was performed as in 24-well plates, incubated until the cells attained monolayer confluency and fixed for immunostaining.

### Electric Cell-substrate Impedance Sensing (ECIS) of Endothelial Barrier Function

Using ECIS, the response of endothelial barrier to the stimulus can be assessed by continuously recording changes in transendothelial resistance (TEER) [28, 29]. Endothelial barrier function was detected in real time using the ECIS® Z-theta system (Applied Biophysics, http://www.biophysics.com/barrierfunction.php) with associated software v.1.2.126 PC as follows: after equilibrating with EBM-2 basal medium (Clonetics, Lonza, USA) for 24 h, 8W10E+ arrays were coated with rat tail collagen type I (diluted 0.01% in sterile pyrogen-free water; BD Biosciences, USA) for 12 h. A monodisperse suspension of HCAEC in EGM-2 MV medium (Clonetics, Lonza, USA) supplemented with 5% FBS was added to the arrays at a density of 80,000–90,000 cells/well and incubated up to 24 h to form a uniform dense monolayer (S1 Fig). Treatments were carried out in fresh medium with or without stimuli. Resistance of HCAEC monolayers was continuously measured (every 5 min) in Ohms at multiple frequencies ranging from 62.6 Hz to 64 kHz. Each of the eight wells of the 8W10E+ arrays contain 40 electrodes that trace the cells at 40 different locations in each well. The measurements of duplicate wells were grouped and averaged to plot as a single curve with error bars representing mean ± SD.

### Automated Cell Migration Assay Using ECIS

The non-invasive ECIS assisted wound healing assay makes a highly reproducible wound with a specific diameter of 250 μm without damaging cells in the surrounding region in 8W1E arrays [30]. It was used for studying cell migration and was performed as follows: equilibration and collagen coating of 8W1E single electrode arrays were performed as described above for 8W10E+ arrays. A monodisperse suspension of HCAEC in EGM-2 MV medium (Clonetics, Lonza, USA) supplemented with 5% FBS was added to the arrays at a density of 80,000–90,000 cells/well and incubated up to 24 h to form a uniform dense monolayer. Treatments were carried out as described above for barrier function assays. Three hours after treatment, wounding was activated with default conditions for 8W1E arrays (1400 μA, 60000 Hz for 20 sec). This killed the treated cells adhering to the electrode surface in 8W1E slides to create a wound. After wounding, impedance was continuously recorded to observe the migration of cells to the wounded area to form a confluent HCAEC monolayer.

### Statistical Analysis

Data were analyzed using GraphPad Prism software version 5.04 (GraphPad Software, San Diego, CA). An unpaired 2-tailed Student’s *t*-test or for comparison of data among groups, 1-way ANOVA followed by the Newman-Keuls test was used and differences were considered statistically significant at *P*<0.05.

## Results

### Gene expression changes in IL-4 treated adult human VEC

To identify all genes regulated by IL-4 treatment in HCAEC, we performed dual channel oligonucleotide based transcriptome profiling. The transcriptome profile of HCAEC treated with IL-4 for 8 h was compared to that of non-treated cells using whole human genome oligomicroarrays. The preprocessed gene expression data was analyzed by GeneSpring software to identify the genes in treated cells that showed a minimum two fold change in expression compared with non-treated cells. IL-4 regulated 2747 genes, of which 1395 genes were upregulated and 1352 genes were down regulated. *PMCH*, *HAS3*, *CCL26*, *OTOGL*, *TMTC1*, *CH25H*, *VCAM1*, *DKK2*, *SLC10A7*, *MASP1*, *COL3A1* and *WNT5A* were the genes most upregulated by IL-4. The top one hundred genes upregulated by IL-4 in HCAEC are listed in S1 Table. *LRRC14B*, *DNAH2*, *TMEM191B*, *FAM47B*, *GPR116*, *TTC9*, *NOG*, *OR13C8*, *GPRC6A*, *CSN3*, *TMEM236* and *PRKG2* were the genes most downregulated by IL-4. The top one hundred genes downregulated by IL-4 in HCAEC are listed in S2 Table.

**Fig 1:**
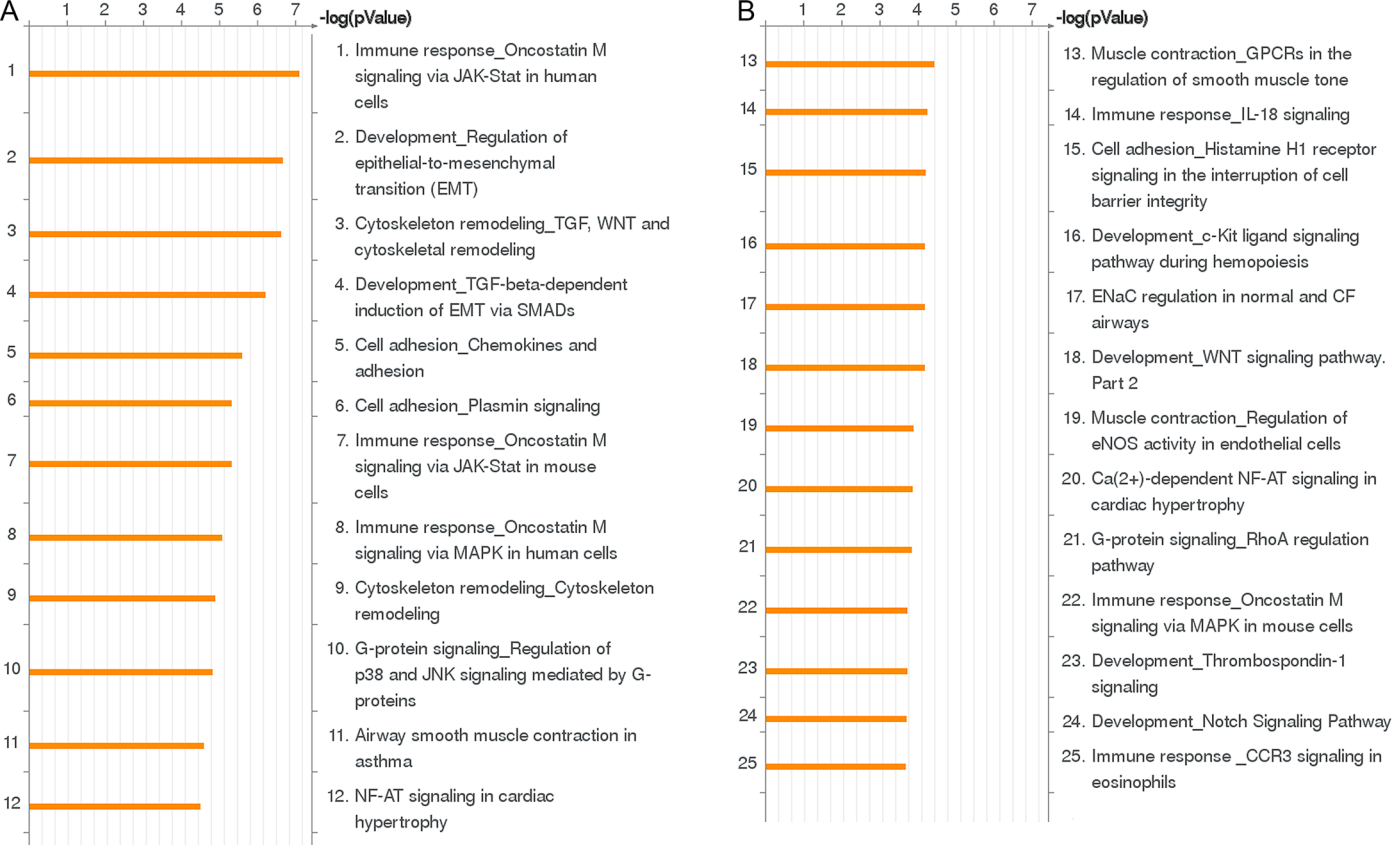
The twenty five biological pathways most significantly (*P*<0.05) regulated by IL-4 treatment in HCAEC. (A, B) Pathways represented as histograms are ranked by the–log value (*P* value). Length of histogram corresponds to the number of genes associated with that specific pathway.

All differentially regulated genes contained in the preprocessed microarray data were subjected to GO analyses using MetaCore™ GeneGO software. Using this tool, genes regulated at least two fold in their expression were clustered on the basis of their function to generate statistically significant cellular pathways. Among the top twenty five biological pathways regulated by IL-4, six were associated with immune responses (Fig 1A and 1B). The ‘Immune response_Oncostatin M signaling via JAK-Stat in human cells’ is the pathway most significantly regulated by IL-4 treatment in HCAEC (Fig 1A). Genes of this pathway regulated by IL-4 (e.g. *CCL2*, *IL6ST*, *MMP1* and *VEGFA*; Table 1) are mainly associated with immune responses involving monocyte-endothelial interactions and endothelial permeabilization. The ‘Development_Regulation of epithelial-to-mesenchymal transition (EMT)’ is the second most significant biological process for IL-4 in HCAEC (Fig 1A). Among the genes of this pathway regulated by IL-4 (Table 1), *DLL4* and *PDGFB* are involved in cell proliferation and migration, *SNAI1* and *ZEB1* are involved in promoting the transition of cells from epithelial to mesenchymal state, *SNAI2* is involved in repressing the expression of cellular junctional proteins and *Wnt5A* is involved in cell migration and inflammation.

**Table 1:** Key genes regulated by IL-4 in ‘Immune response_Oncostatin M signaling via JAK-Stat in human cells’, ‘Development_Regulation of epithelial-to-mesenchymal transition (EMT)’ and ‘Cytoskeleton remodeling_TGF, Wnt and cytoskeletal remodeling’ pathways in HCAEC.

Gene Symbol	Accession	Sequence Description	Regulation
CAV1^c^	NM_001172895	Homo sapiens caveolin 1	Down
CCL2^a^	NM_002982	Homo sapiens chemokine (C-C motif) ligand 2	Up
CFL1^c^	NM_005507	Homo sapiens cofilin 1	Down
CCND1^a^^,^^c^	NM_053056	Homo sapiens cyclin D1	Down
DLL4^b^	NM_019074	Homo sapiens delta-like 4	Down
FZD4^b^^,^^c^	NM_012193	Homo sapiens frizzled class receptor 4	Up
FZD8^b^^,^^c^	NM_031866	Homo sapiens frizzled class receptor 8	Up
FOXO3^c^	NM_001455	Homo sapiens forkhead box O3	Up
HEY1^b^	NM_001040708	Homo sapiens hes-related family bHLH transcription factor with YRPW motif 1	Down
IL6ST^a^	NM_001190981	Homo sapiens interleukin 6 signal transducer	Up
JAG1^b^	NM_000214	Homo sapiens jagged 1	Up
KDR^c^	NM_002253	Homo sapiens kinase insert domain receptor	Down
LIFRα^a^	NM_002310	Homo sapiens leukemia inhibitory factor receptor alpha	Up
LIMK2^c^	NM_001031801	Homo sapiens LIM domain kinase 2	Down
MAPK13^c^	NM_002754	Homo sapiens mitogen-activated protein kinase 13	Down
MAP2K3^c^	NM_002756	Homo sapiens mitogen-activated protein kinase kinase 3	Down
MMP1^a^	NM_002421	Homo sapiens matrix metallopeptidase 1	Up
MMP7^c^	NM_002423	Homo sapiens matrix metallopeptidase 7	Up
MYLK^c^	NM_053025	Homo sapiens myosin light chain kinase	Down
MYLK2^c^	NM_033118	Homo sapiens myosin light chain kinase 2	Down
PDGFβ^b^	NM_002608	Homo sapiens platelet-derived growth factor beta polypeptide	Down
SNAI1^b^	NM_005985	Homo sapiens snail homolog 1	Down
SNAI2^b^	NM_003068	Homo sapiens snail homolog 2	Down
TGFβ2^b^	NM_003238	Homo sapiens transforming growth factor, beta 2	Up
TGFβ3^b^	NM_003239	Homo sapiens transforming growth factor, beta 3	Up
VEGFA^a^^,^^c^	NM_001025366	Homo sapiens vascular endothelial growth factor A	Up
WNT5A^b^^,^^c^	NM_003392	Homo sapiens wingless-type MMTV integration site family, member 5A	Up
ZEB1^b^	NM_001128128	Homo sapiens zinc finger E-box binding homeobox 1	Down

^a^Genes regulated by IL-4 in ‘Immune response_Oncostatin M signaling via JAK-Stat in human cells’ pathway.

^b^Genes regulated by IL-4 in ‘Development_Regulation of epithelial-to-mesenchymal transition (EMT)’ pathway.

^c^Genes regulated by IL-4 in ‘Cytoskeleton remodeling_TGF, Wnt and cytoskeletal remodeling’ pathway.

**Fig 2:**
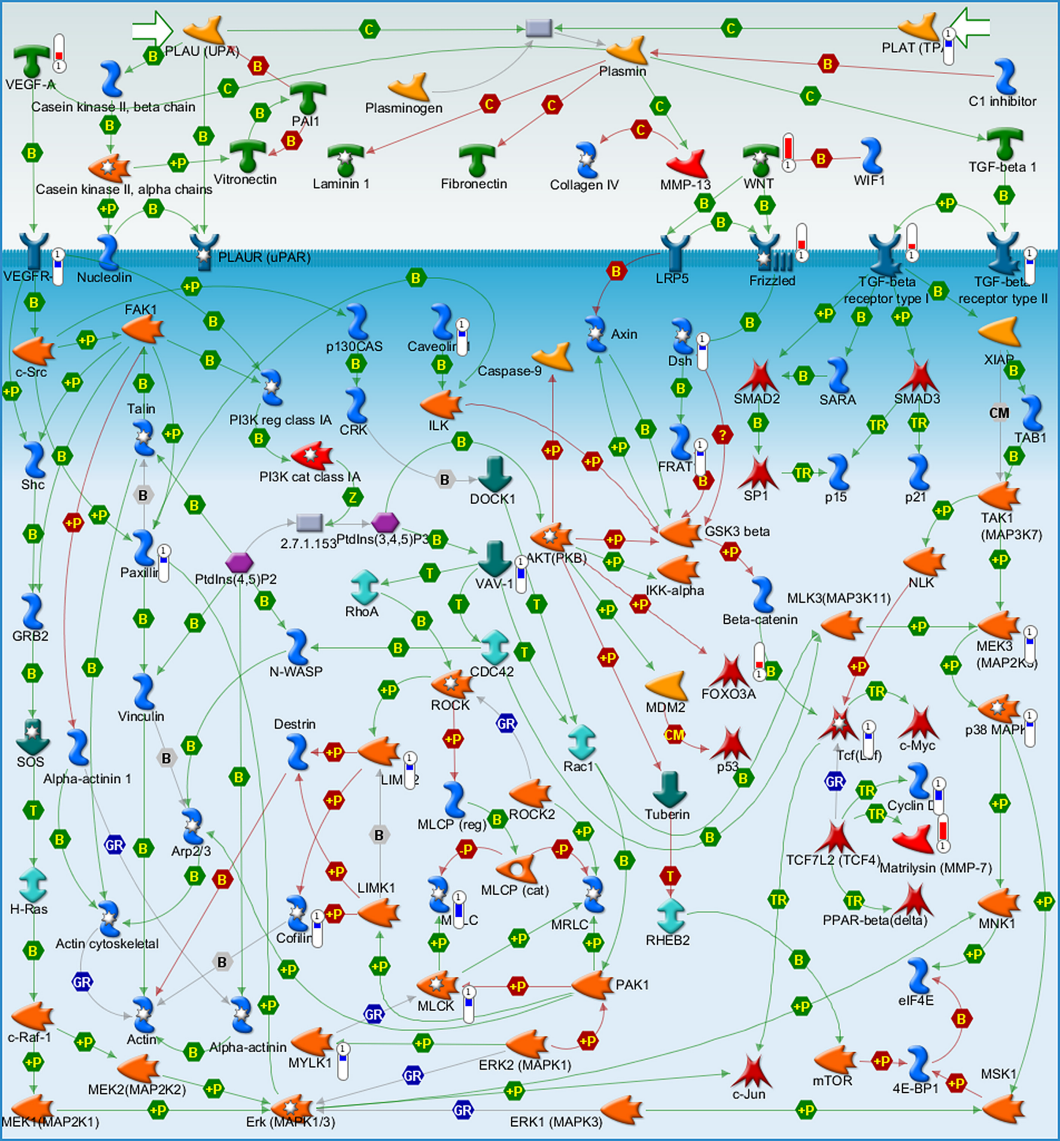
Metacore™ map showing the signaling context of the genes contained in the ‘Cytoskeleton remodeling_TGF, Wnt and cytoskeletal remodeling’ pathway. Genes regulated by IL-4 are marked by red and blue thermometer icons representing upregulated and downregulated gene expression respectively.

The ‘Cytoskeleton remodeling_TGF, Wnt and cytoskeletal remodeling’ pathway is the third most significant biological process for IL-4 in HCAEC (Fig 1A). Among the genes regulated in this pathway are *Wnt5A*, *Wnt1*, *VEGFA*, *LIMK2* and *CFL1* (Table 1). The MetaCore™ map of this pathway showing these genes within their signaling context indicates that IL-4 upregulates Wnt as most significant ligand for the cytoskeleton remodeling (Fig 2, upper right quarter), and causes downstream regulation of LIMK2 and CFL1 (Fig 2, lower left quarter). *LIMK2* and *CFL1* are responsible for actin polymerization. Among the other genes of this pathway are *KDR* and *FZDs* (Fig 2, Table 1), which are involved in VEGF and Wnt signaling.

Data from our study confirm previous findings that IL-4 upregulatesVCAM-1 [10–12], IL-6 [12–14], MCP [12, 14] and LIFR [12] in VEC (S1 Table). Thus, it corroborates the proposed role of IL-4 as an ‘alternative inflammatory’ cytokine. With respect to transcription factors mediating IL-4 induced gene expression in VEC, the present study demonstrates that IL-4 upregulates the expression of genes encoding FOSB (a member of the activator protein-1 family), GATA3 and EGR1 (S3 Table).

### Confirmation of Wnt5A expression

**Fig 3:**
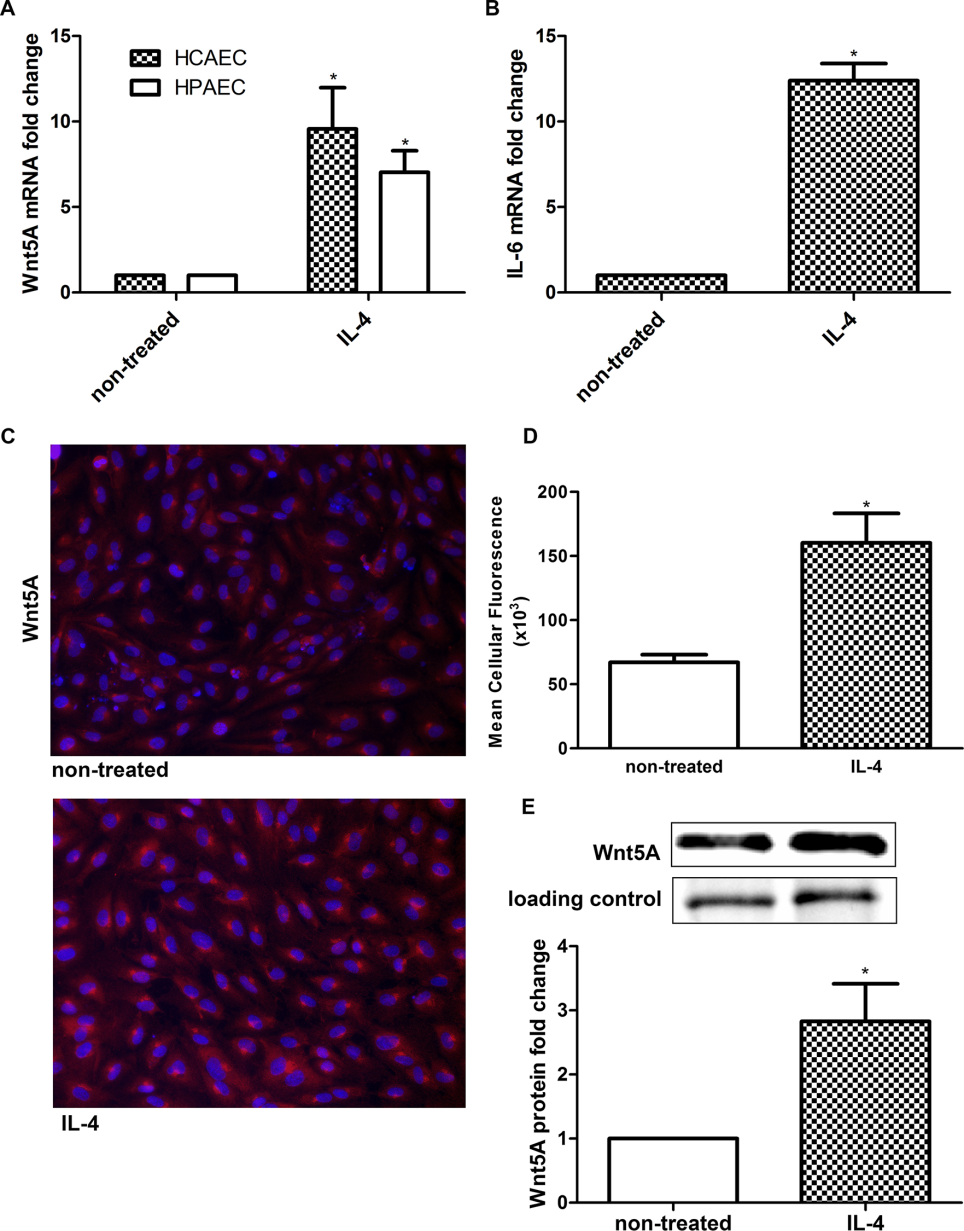
Expression of Wnt5A in IL-4 treated HCAEC. Fold changes in the expression of (A) Wnt5A mRNA in 8 h IL-4 treated HCAEC and HPAEC and (B) IL-6 mRNA in 8 h IL-4 treated HCAEC. Data were obtained from three independent qRT-PCR experiments run with duplicate samples and expressed as the mean ± SEM. *P<0.05. (C) Wnt5A protein expression in non-treated and IL-4 treated HCAEC. Wnt5A is immunostained (red), nuclei (DAPI, blue). Photomicrographs were acquired using a Zeiss Axioskope equipped with AxioCam MRm digital camera and AxioVision Rel.4.6 software. Original magnification, 200×. (D) Mean cellular fluorescent intensity of Wnt5A. Data represent mean ± SD from five different regions. *P<0.05. The experiment was repeated five times, with analogous results. (E) Immunoblot of Wnt5A induction in IL-4 treated HCAEC. In-gel stained 50 kDa band, served as loading control and for densitometry normalization. Left band, non-treated cells; right band, IL-4 treated cells. Data are mean ± SEM from three independent experiments. *P<0.05.

Since Wnt5A was previously identified as an inflammatory mediator, and expression of the gene is shown to be significantly upregulated by IL-4 in microarray analysis, we measured Wnt5A mRNA levels in HCAEC treated with IL-4 for 8 h using qRT-PCR. Consistent with the microarray data, IL-4 significantly upregulated Wnt5A mRNA levels (Fig 3A). The induction of IL-6 mRNA was considered as indication of positive IL-4 stimulation of HCAEC (Fig 3B). To confirm that IL-4 induced Wnt5A expression is not only specific for HCAEC but also for endothelial cells from different vascular beds, we measured Wnt5A mRNA level in IL-4 treated HPAEC. Consistent with findings obtained in HCAEC, 8 h treatment with IL-4 significantly upregulated Wnt5A in HPAEC (Fig 3A). We further confirmed Wnt5A induction at the protein level by immunofluorescence staining and immunoblot. In accordance with qRT-PCR and microarray data, treatment with IL-4 notably increased the levels of Wnt5A protein in HCAEC (Fig 3C–3E).

### LIM kinase (LIMK) 2 and Cofilin-1 (CFL1) are phosphorylated in IL-4 treated HCAEC

**Fig 4:**
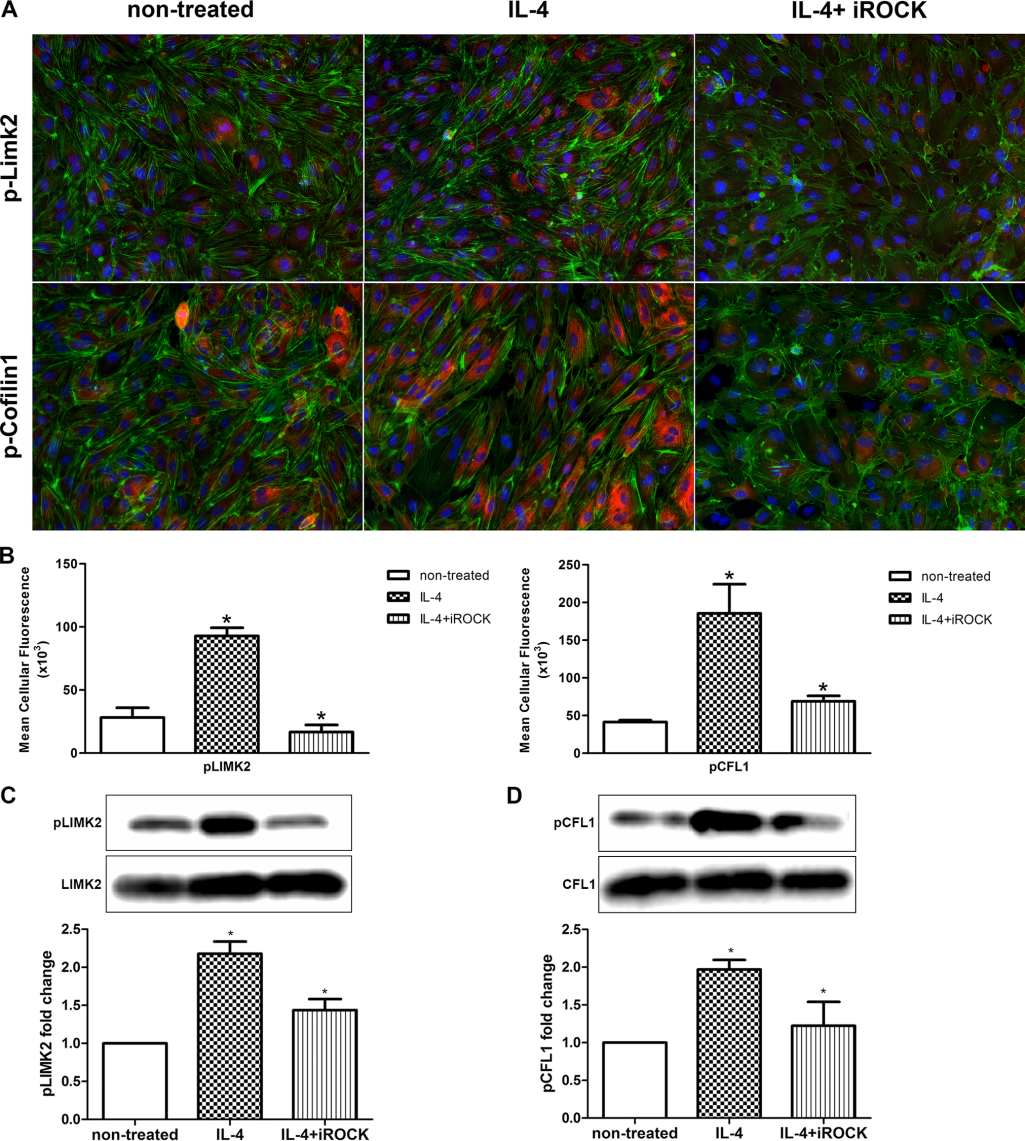
Phosphorylation of LIMK2 and CFL1 in IL-4 treated HCAEC. (A) pLIMK2 (pT505) and pCFL1 (pCofilin-1, pS3) were immunostained (red) in HCAEC treated with IL-4 either alone or in combination with Y-27632. F-actin (fluorescent phalloidin, green) and nuclei (DAPI, blue) were also stained. Photomicrographs were acquired using a Zeiss Axioskope equipped with AxioCam MRm digital camera and AxioVision Rel.4.6 software. Original magnification, 200×. (B) Mean cellular fluorescent intensities of pLIMK2 and pCFL1. Data represent mean ± SD from five different regions. *P<0.05. The experiment was repeated three times, with analogous results. (C, D) Immunoblots of pLIMK2, LIMK2, pCFL and CFL in HCAEC treated with IL-4 either alone or in combination with Y-27632. Left band, non-treated cells; middle band, IL-4 treated cells; right band, IL-4+Y-27632 treated cells. Data are mean ± SEM from three independent experiments. *P<0.05.

GO analysis of gene expression data shows that IL-4 regulated the *LIMK2* and *CFL1* genes, which are involved in cytoskeleton remodeling (Fig 2, Table 1). LIMK2, a serine/threonine/tyrosine kinase, is a downstream target of ROCK. When phosphorylated by the activated ROCK, LIMK2 phosphorylates the actin depolymerization factor CFL1 [31–33]. Therefore, we investigated whether LIMK2 and CFL1 proteins are phosphorylated following IL-4 treatment in HCAEC. Immunofluorescence staining and immunoblot employing specific antibodies were used to detect and quantify the phosphorylated forms of LIMK2 (pLIMK2) and CFL1 (pCFL1). Compared to non-treated cells, pLIMK2 and pCFL1 levels were markedly increased in HCAEC treated with IL-4 for 1 h and 4 h respectively (Fig 4A–4D). At earlier (30 min, 2 h) or later (8 h) time points, no significant change in phosphorylation of LIMK2 and CFL1, respectively, was observed (data not shown). Combining IL-4 with the ROCK inhibitor Y-27632 significantly suppressed the phosphorylation of LIMK2 and CFL1 compared with IL-4 alone (Fig 4A–4D).

### IL-4 induced stress fiber formation can be decreased by Wnt antagonist

**Fig 5:**
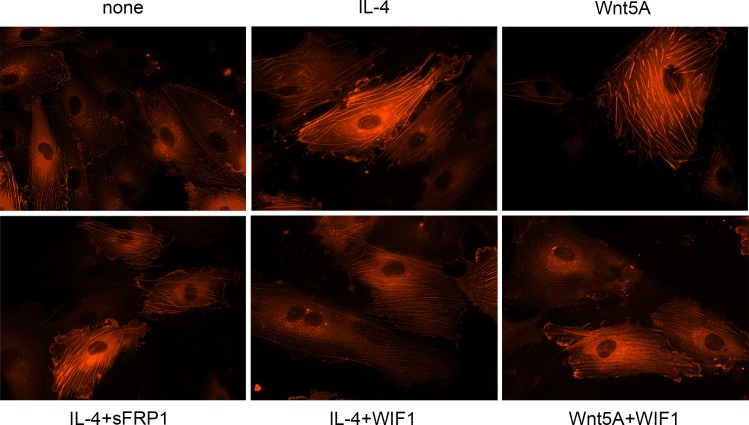
Stress fiber formation in IL-4 treated HCAEC. Live actin-RFP staining showing the formation of actin stress fibers in HCAEC treated with IL-4 or Wnt5A in the absence or presence of sFRP1 and WIF1. Expression of de novo synthesized RFP-actin after transfection as outlined in methods was visually observed up to 24 h. Photomicrographs of stress fiber formation were taken randomly at 12 h after stimulation with IL-4 and Wnt5A alone or in the presence of sFRP1 and WIF1 using Zeiss Axio Observer.Z1 equipped with AxioCam MRm digital camera and ZEN 2012 software. Original magnification, 400×. Independent identical experiments in triplicates were repeated at least three times, with analogous results.

Since inactivation of CFL1 by phosphorylation prevents actin depolymerization resulting in F-actin stress fiber formation [32–34], and IL-4 treatment induced its phosphorylation, we next investigated whether IL-4 causes stress fiber formation. Alterations in actin organization and stress fiber formation were visualized by imaging actin-RFP in IL-4 treated live HCAEC. Actin-RFP showed only a few thin stress fibers in non-treated HCAEC whereas formation of thick actin stress fibers were visible in IL-4 treated cells (Fig 5).

Functional clustering of the IL-4 regulated transcriptome showed upregulation of Wnt5A, and this occurs upstream of LIMK2 and CFL1 regulation in the cytoskeleton remodeling process (Fig 2). Having verified upregulated Wnt5A expression and increased stress fiber formation through LIMK2 and CFL1 phosphorylation in IL-4 treated HCAEC, we next investigated the role of Wnt5A in IL-4 induced stress fiber formation using Wnt antagonists. As Frizzleds (Fzds) have long been considered the receptors for Wnts, and transcriptome profiling in this study showed upregulation of some *Fzd* genes by IL-4 in HCAEC, we first attempted to block Wnt5A/Fzd signaling using sFRP1. Treating HCAEC with IL-4 in the presence of sFRP1 did not influence actin stress fiber formation (Fig 5). As Ryk is described as an alternative receptor for Wnts [35], we next tested whether inhibiting Wnt5A/Ryk interaction by using WIF1 has an effect on IL-4 induced stress fiber formation. Stress fiber formation was notably suppressed when HCAEC were treated with a combination of IL-4 and WIF1 (Fig 5). HCAEC showed increased stress fiber formation upon Wnt5A treatment, however this was notably suppressed after treatment with a combination of Wnt5A and WIF1 (Fig 5).

### IL-4 disrupts the assembly of VE-cadherin in inter-endothelial junctions (IEJs)

**Fig 6:**
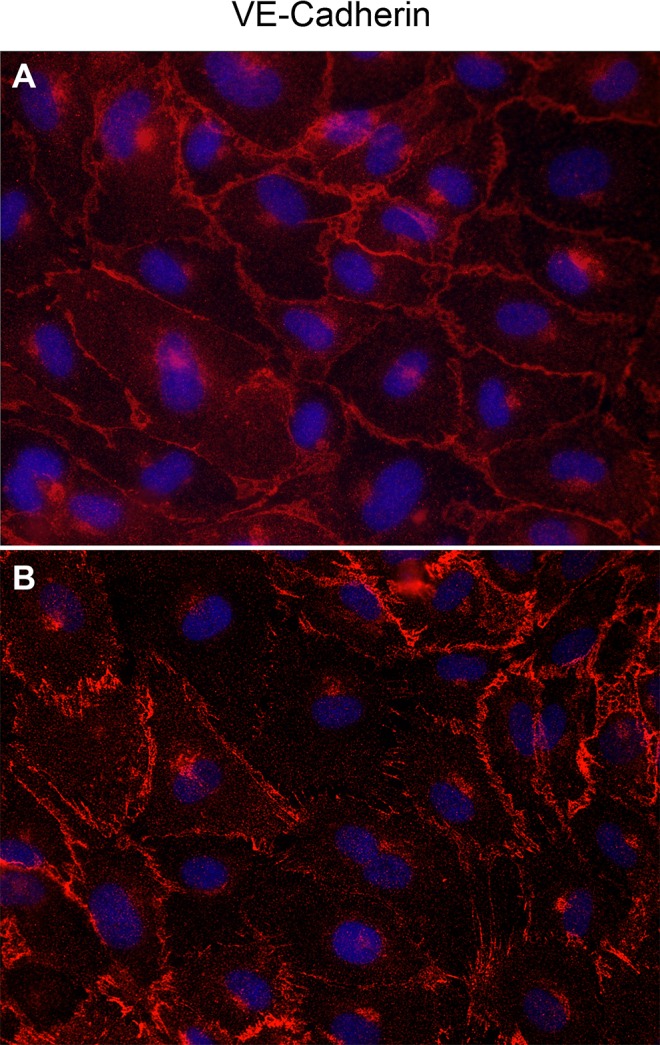
Assembly of VE-cadherin in IL-4 treated HCAEC. VE-cadherin immunostained (red) in HCAEC either non-treated (A) or treated with IL- 4 for 8 h (B). Nuclei (DAPI, blue). Photomicrographs were acquired using a Zeiss Axioskope equipped with AxioCam MRm digital camera and AxioVision Rel.4.6 software. Original magnification, 400×.

Actin stress fiber formation leads to disassembly of VE-cadherin in IEJs and causes intercellular gap formation in endothelial monolayers [36]. Since IL-4 induced stress fiber formation, we next investigated if IL-4 affected VE-cadherin assembly in HCAEC. Immunofluorescence staining showed continuous distribution of VE-cadherin along the cellular periphery, and tight intercellular contacts in non-treated HCAEC monolayers (Fig 6A). In contrast, IL-4 treated HCAEC monolayers showed small inter-endothelial gaps with marked loss of VE-cadherin at intercellular regions (Fig 6B).

### IL-4 induced endothelial hyperpermeability is decreased by silencing Wnt5A

**Fig 7:**
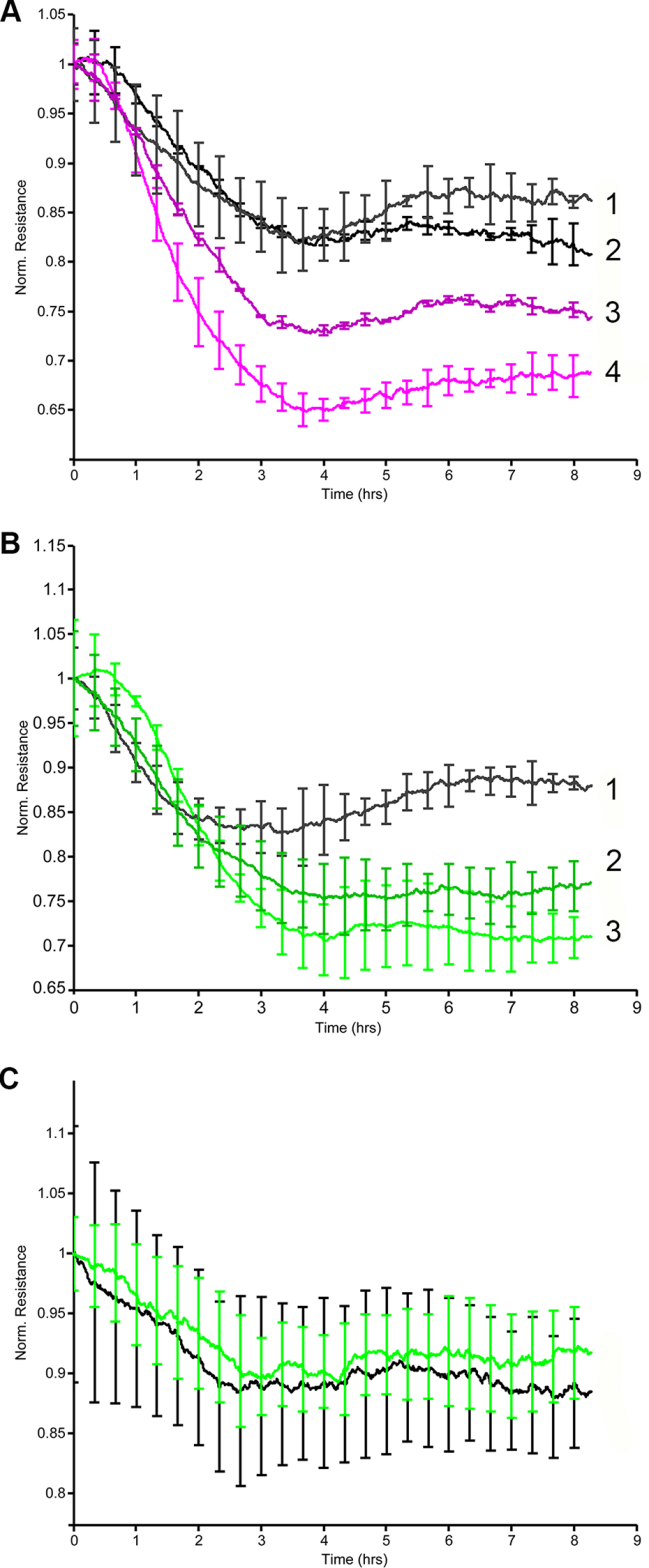
ECIS barrier function assays showing the effect of Wnt5A in IL-4 induced hyperpermeability of HCAEC. Uniform confluent monolayers of HCAEC cultured in stabilized and collagen coated ECIS 8W10E+ arrays were treated with IL-4. TEER of HCAEC monolayers was measured in Ohms continuously every 5 min at multiple frequencies ranging from 62.6 Hz to 64 kHz, normalized to its value at time zero and plotted with respect to time. Stimulations conducted in duplicate wells were grouped and averaged to plot a single curve with error bars representing mean ± SD. Figures shown depict the resistance measurements conducted at 4000 Hz and represent four independent experiments. (A) 1, non-treated Wnt5A-siRNA transfected HCAEC; 2, non-treated non-transfected HCAEC; 3, IL-4 treated Wnt5A-siRNA transfected HCAEC; 4, IL-4 treated non-transfected HCAEC. (B) 1, non-treated negative control siRNA transfected HCAEC; 2, IL-4 treated negative control siRNA transfected HCAEC; 3, IL-4 treated non-transfected HCAEC. (C) Black, non-treated; green, IL-6.

Stress fiber formation and IEJ disruption accompanied by inter-endothelial gap formation impairs barrier function and increases permeability of endothelial monolayers [36]. Since IL-4 upregulated Wnt5A, and inhibiting Wnt5A with WIF1 decreased IL-4 induced stress fiber formation in HCAEC, we investigated whether Wnt5A mediated IL-4 induced endothelial hyperpermeability. Alterations in TEER of tight HCAEC monolayers cultured in 8W10E+ slides were recorded in real time by ECIS. Treatment with IL-4 significantly reduced the TEER of HCAEC monolayers to alternating current (AC). Notably, IL-4 induced alterations in TEER became apparent 3 h after treatment with IL-4 and persisted for more than 8 h (Fig 7A).

Next, we tested whether silencing Wnt5A improves the barrier function of IL-4 treated HCAEC monolayers. HCAEC were transfected with siRNA against Wnt5A. qRT-PCR showed that Wnt5A mRNA expression was approximately 60% lower in Wnt5A-siRNA transfected cells than in cells transfected with negative control siRNA (S2A Fig). Accordingly, immunofluorescence staining showed decreased Wnt5A protein expression in Wnt5A-siRNA transfected HCAEC compared with negative control siRNA transfected cells (S2B and S2C Fig).

Upon IL-4 treatment, Wnt5A silenced HCAEC showed significantly increased TEER compared with non-transfected HCAEC (Fig 7A). In HCAEC transfected with negative control siRNA, the response to IL-4 was not altered (Fig 7B).

Since IL-4 upregulated IL-6 in HCAEC in the present study (Fig 3B), and IL-6 has been reported to increase permeability of HUVEC [37] and bovine vascular endothelial cells (BVEC) [38], we also investigated whether IL-6 increased permeability of HCAEC monolayers. Treatment with IL-6 did not alter the TEER of HCAEC monolayer to AC, thereby indicating that IL-6 does not influence the permeability characteristics of HCAEC (Fig 7C).

### IL-4 impairs migration of HCAEC, which can be restored by ROCK inhibition

**Fig 8:**
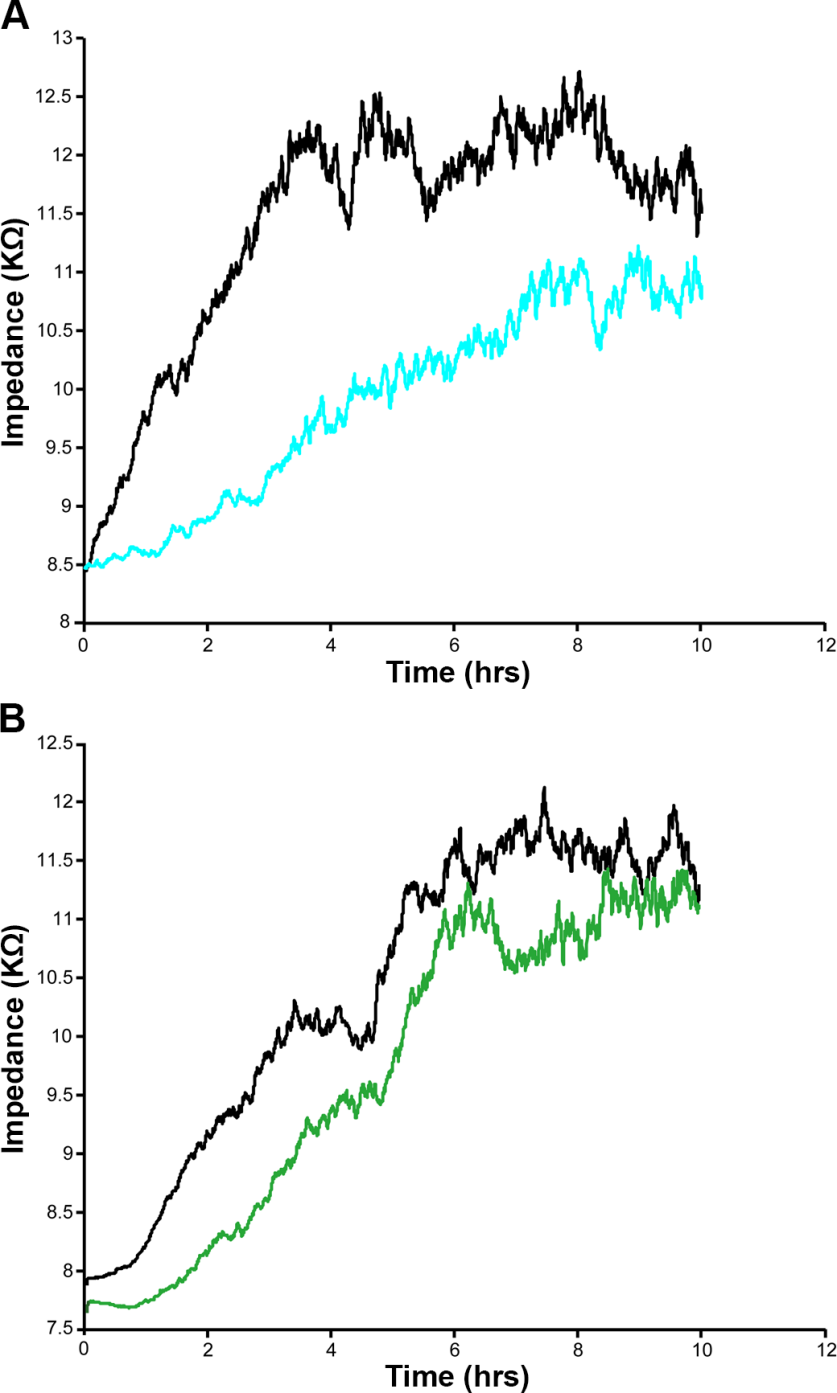
Automated wound healing assay showing decreased motility of IL-4 treated HCAEC. Uniform confluent monolayers of HCAEC cultured in stabilized and collagen coated ECIS 8W1E cultureware were treated with IL-4 either alone or in combination with Y-27632. Three hours after treatment, wells were subjected to an elevated electric field to create a wound. Measurements were started immediately after wounding. Impedance of HCAEC monolayer was continuously measured in Ohms every 5 min at multiple frequencies ranging from 62.6 Hz to 64 kHz and plotted with respect to time. Representative figures depict the impedance of wells after wounding was applied. Figures shown depict the measurements conducted at 4000 Hz and represent four independent experiments. (A) Black, non-treated; Blue, IL-4. (B) Black, control with Y27632; Green, IL-4 + Y27632.

Since IL-4 increased the formation of actin stress fibers and caused inter-endothelial gap formation, we investigated whether IL-4 would also affect the motility of HCAEC. In an ECIS supported wound healing and cell migration assay, IL-4 treated HCAEC exhibited a lower impedance and required a longer time to heal the wound compared with non-treated cells (Fig 8A). This indicates that IL-4 significantly reduces endothelial cell motility, causing delayed wound closure.

The present study shows that IL-4 induced phosphorylation of LIMK2 and CFL1 can be decreased by inhibiting ROCK. Since ROCK regulates cytoskeleton remodeling, cell–cell adhesion and cell motility [33], we investigated whether inhibiting ROCK prevents IL-4 induced impairment of HCAEC motility. Cells treated with a combination of IL-4 and the ROCK inhibitor Y-27632 showed signifcantly higher motility rates and faster wound closure than cells treated with only IL-4 (Fig 8B).

### IL-4 upregulates Wnt5A expression in human macrophages

**Fig 9:**
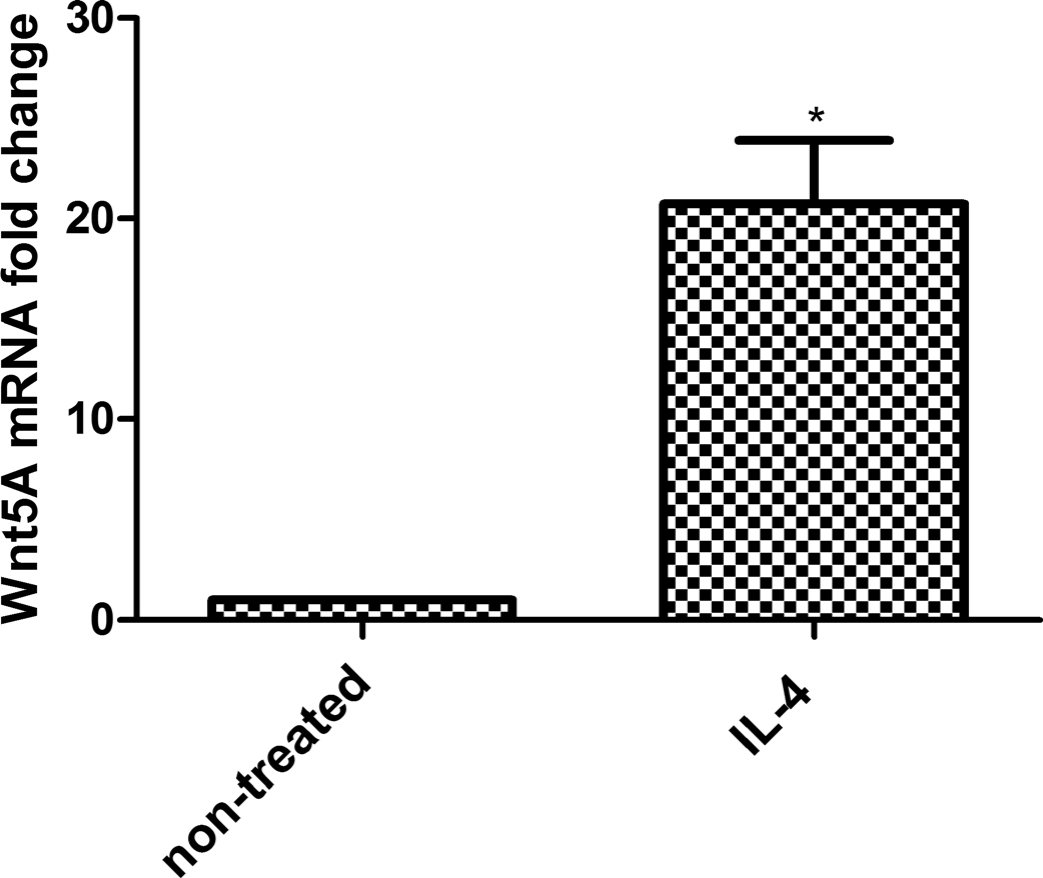
Wnt5A expression in IL-4 activated human macrophages. Fold changes in the expression of Wnt5A mRNA in IL-4 treated human macrophages. Data were obtained from two independent qRT-PCR experiments run with duplicate samples using RNA isolated from macrophages from four different donors and expressed as the mean ± SEM. *P<0.05 using an unpaired Student’s *t*- test.

Since macrophages also play a pivotal role in Th2 inflammation, we asked whether Wnt5A expression is also upregulated in IL-4 activated human M2 type macrophages. Treatment with IL-4 significantly upregulated Wnt5A mRNA expression in macrophages (Fig 9).

## Discussion

VEC play an important role in maintaining vascular homeostasis. During inflammation, soluble mediators secreted by activated immune cells paracrinically act on VEC and significantly alter their functions to confer ‘inflamed’ or ‘activated’ phenotypes on them. IL-4 is a potent Th2 cytokine known to cause activation of VEC and induce endothelial barrier dysfunction [21]. However, the exact mechanisms responsible for IL-4 dependent endothelial dysfunction in VEC remained unclear. In the present study, we detected all genes regulated by paracrine IL-4 signaling in adult VEC by whole human genome microarray based transcriptome profiling (Fig 1, S1 and S2 Tables).

This is the first study to demonstrate that IL-4 significantly upregulates Wnt5A expression in adult VEC. A previous study demonstrated that HUVEC treated with a combination of TNF-α, IL-1 and IL-8 upregulated the expression of Wnt5A mRNA [39]. Wnt5A is a non-canonical Wnt ligand, recently identified as a pro-inflammatory mediator of macrophage activation in vascular inflammation [24]. Wnt5A has been demonstrated to enhance the permeability of HUVEC monolayers in a ^14^C sucrose permeability test [40] and IL-4 caused morphological changes and induced inter-endothelial gap formation in HUVEC [17]. Furthermore IL-4 has been demonstrated to induce hyperpermeability of HUVEC [21]. Inter-endothelial gap formation caused by cytoskeletal rearrangements impairs the barrier functions of endothelial monolayers and is the principal pathway causing endothelial hyperpermeability and subsequent edema formation in inflammation [41]. Endothelial hyperpermeability accounts for increased efflux of plasma through microvessel walls into neighboring tissues, leading to the formation of protein rich tissue edema. Edema formation resulting from vascular leakage is an important consequence of allergic inflammation [21] and a most common side effect of IL-4 therapy in human cancer patients [42]. Addressing a potential role for Wnt5A in IL-4 induced endothelial cytoskeleton remodeling, GO analyses for the current study point to Wnt5A as a potential ligand for the ‘Cytoskeleton remodeling_TGF, Wnt and cytoskeletal remodeling’ pathway (Fig 2). A prominent role for Wnt5A in mediating IL-4 induced cytoskeleton remodeling is evident from our observation that IL-4 induced actin stress fiber formation can be suppressed by blocking Wnt5A signaling (Fig 5). Further proof for the involvement of Wnt5A is provided by functional experiments demonstrating that the enhanced permeability of HCAEC monolayers upon IL-4 treatment can be significantly decreased by silencing Wnt5A expression (Fig 7A). Our findings support the crucial role of Wnt5A in stress fiber formation and inter-endothelial gap formation, leading to impaired barrier function in IL-4 treated HCAEC. However, it must be noted that targeting Wnt5A does not completely prevent IL-4 induced stress fiber formation (Fig 5) and endothelial hyperpermeability (Fig 7A). Therefore, it cannot be excluded that IL-4 contributes to endothelial hyperpermeability also by other yet unknown mechanisms.

Wnt5A signals mainly through Fzd receptors but can also function through Ryk, a member of the family of atypical receptor tyrosine-protein kinases (RTKs). Ryk consists of an extracellular Wnt-binding domain, a PDZ binding motif and an intracellular inactive tyrosine kinase domain. Its Wnt-binding domain is homologous to the extracellular Wnt antagonist, WIF protein. sFRP1 is another Wnt antagonist that contains a frizzled like cysteine-rich domain (CRD) homologous to the extracellular Wnt-binding domains of Fzd receptors [35]. The availability of the receptor, presence of Wnt antagonists and the cellular environment determines which receptors Wnts engages and the signals that are generated. The involvement of Ryk, but not Fzd receptors, in IL-4 induced Wnt5A signaling in HCAECs is obvious from our observation that stress fiber formation was prevented in the presence of WIF1 but not sFRP1 (Fig 5). Others have demonstrated that Wnt5A/Ryk signaling through Rho-kinase activation inhibits axon growth in rats [43]. We propose that in HCAEC, Wnt5A signaling through Ryk receptor activates ROCK that in turn phosphorylates LIMK2. Activated pLIMK2 in turn phosphorylates CFL1 that is then deactivated, and as a consequence, allows actin fiber polymerization. This disrupts VE-cadherin assembly in IEJs leading to the formation of inter-endothelial gaps, as we have observed in the present study (Fig 6B). The involvement of ROCK as a downstream effector of Wnt5A/Ryk signaling in HCAEC is very well supported by the observations that phosphorylation of LIMK2 and CFL1 following IL-4 treatment can be decreased by inhibiting ROCK (Fig 4). The function of ROCK in IL-4 induced cytoskeleton remodeling is further evident from the standardized ECIS cell migration assay that shows improved migratory and wound healing capacity of cells treated with a combination of IL-4 and ROCK inhibitor (Fig 8A and 8B). Phosphorylation of LIMK and CFL at the indicated time points is also in line with the observations that IL-4 induced LIMK-CFL1 downstream signaling leads to the functional effects occurring at later time points in this study.

Intriguingly, the present study confirms that IL-6 is upregulated by IL-4 in HCAEC but IL-6 does not influence TEER in the ECIS based standardized permeability assay (Fig 7C), even though it increases permeability in HUVEC [37] and BVEC [38]. Further studies are warranted to confirm that IL-6 is not responsible for increased VEC permeability, or whether this effect of IL-6 varies between endothelial cells from different vascular beds and thus depends on endothelial heterogeneity.

The whole genome transcriptome analysis of IL-4 treated HCAEC further revealed induction of vascular endothelial growth factor (VEGF)-A (Table 1, Fig 2). A previous study showed that IL-4 increased the production of VEGF in unstimulated synovial fibroblasts [44]. VEGF-A acts as a proangiogenic factor in wound healing. It enhances endothelial cell migration from pre-existing blood vessels and promotes the recruitment of endothelial progenitor cells (EPCs) from peripheral circulation [45]. Surprisingly, the present study demonstrates an inhibitory effect of IL-4 on endothelial cell migration while upregulating VEGF-A. Even though this contradicts the effects of VEGF in stimulating endothelial cell migration, it could be explained by the observation that IL-4 downregulates expression of VEGFR-2 (Table 1, Fig 2). VEGFR-2 (also known as KDR) is a receptor specific for VEGF [45] and downregulation would block autocrine VEGF-A signaling in endothelial cells. This is a likely feedback mechanism inhibiting angiogenesis. As soluble factors secreted by VEC paracrinically act on other immune cells such as macrophages, it is also possible that VEGF-A produced by VEC interacts with VEGF receptors expressed on other immune cells. This view is well supported by previous studies which demonstrated that VEGF-A acted as a chemoattractant for monocytes expressing VEGF receptor 1 [46–49].

Among the genes highly expressed upon IL-4 treatment of HCAEC (S1 Table), pro-melanin-concentrating hormone (PMCH) has also been described previously as highly induced by activated Th2 cells [50]. IL-4 triggered expression of hyaluronan synthase 3 (HAS3) may facilitate the adhesion and migration of activated immune cells and cancer cells during inflammatory response and cancer metastasis, respectively. Induction of CCL26 by IL-4 has already been described in VEC and it acts as a chemokine for eosinophils [51].

## Conclusions

Our study indicates that Wnt5A is crucially involved in cytoskeleton rearrangement and barrier dysfunction mediated by IL-4 in VEC. We further show that IL-4 induced Wnt5A signals downstream through the ROCK–LIMK2–CFL1 pathway and Ryk is a possible receptor for Wnt5A signaling in VEC. Therapeutic approaches targeting Wnt5A may reduce microvascular leakage and subsequent edema formation associated with IL-4 driven pathophysiological conditions such as allergic and tumor inflammation. Moreover, we show for the first time that IL-4 induces Wnt5A in human M2 type macrophages, which is different to the Toll-like receptor (TLR) mediated Wnt5A induction described previously [24]. Together, this establishes Wnt5A as a component of both classically and alternatively activated macrophages. It can be postulated that Wnt5A secreted by IL-4 activated vascular endothelial cells and macrophages acts paracrinically on vascular smooth muscle cells. Further studies delineating the specific role of Wnt5A in alternatively activated macrophages and vascular smooth muscle cells would provide novel insights into the inflammatory responses in diseases associated with Th2 activity.

## Supporting Information

S1 Fig HCAEC monolayer formation in ECIS arrays. Immediately after seeding HCAEC into 8W10E+ arrays with a density of 80,000–90,000 cells/well, resistance measurements (in Ohms) were started and are shown as normalized resistance (subsequent values were divided by initial values). Increase in resistance over time indicates an increase in the formation of intercellular contacts. The steady state of resistance represents a tight monolayer stage exhibiting stable barrier function. Each single curve represents the resistance measurements conducted in duplicate wells which were grouped and averaged. Error bars of curves represent SD. Figures shown depict the resistance measurements conducted at 4000 Hz. Green and yellow, non-treated.
(DOCX)

S2 Fig Wnt5A knockdown efficiency. (A) Expression levels of Wnt5A mRNA in HCAEC transfected with 5 nM negative (neg.) control siRNA and Wnt5A siRNA. Data were obtained from three independent qRT-PCR experiments run with duplicate samples and expressed as the mean ± SEM. *P<0.005. Representative immunofluorescence staining depicting Wnt5A protein expression (red) in negative control siRNA transfected (B) and Wnt5A siRNA transfected (C) HCAEC. Green: F-actin, Blue: nuclei. Zeiss Axioskope, Magnification 20×.
(DOCX)

S3 Fig Expression of Wnt5A in HCAEC after 4 h stimulation with IL-4. Fold changes in the expression of Wnt5A mRNA in 4 h IL-4 treated HCAEC. Data were obtained from three independent qRT-PCR experiments run with duplicate samples and expressed as the mean ± SEM. *P<0.05.
(DOCX)

S1 Table The top 100 genes upregulated by IL-4 in HCAEC. (DOCX)

S2 Table The top 100 genes downregulated by IL-4 in HCAEC. (DOCX)

S3 Table Regulation of genes coding for transcription factors in IL-4 treated HCAEC. (DOCX)
