# Supplementary figures and images for: IL-4 Causes Hyperpermeability of Vascular Endothelial Cells through Wnt5A Signaling
Source: PLoS One. 2016 May 23;11(5):e0156002. doi: 10.1371/journal.pone.0156002 (PMC4877093; doi:10.1371/journal.pone.0156002)

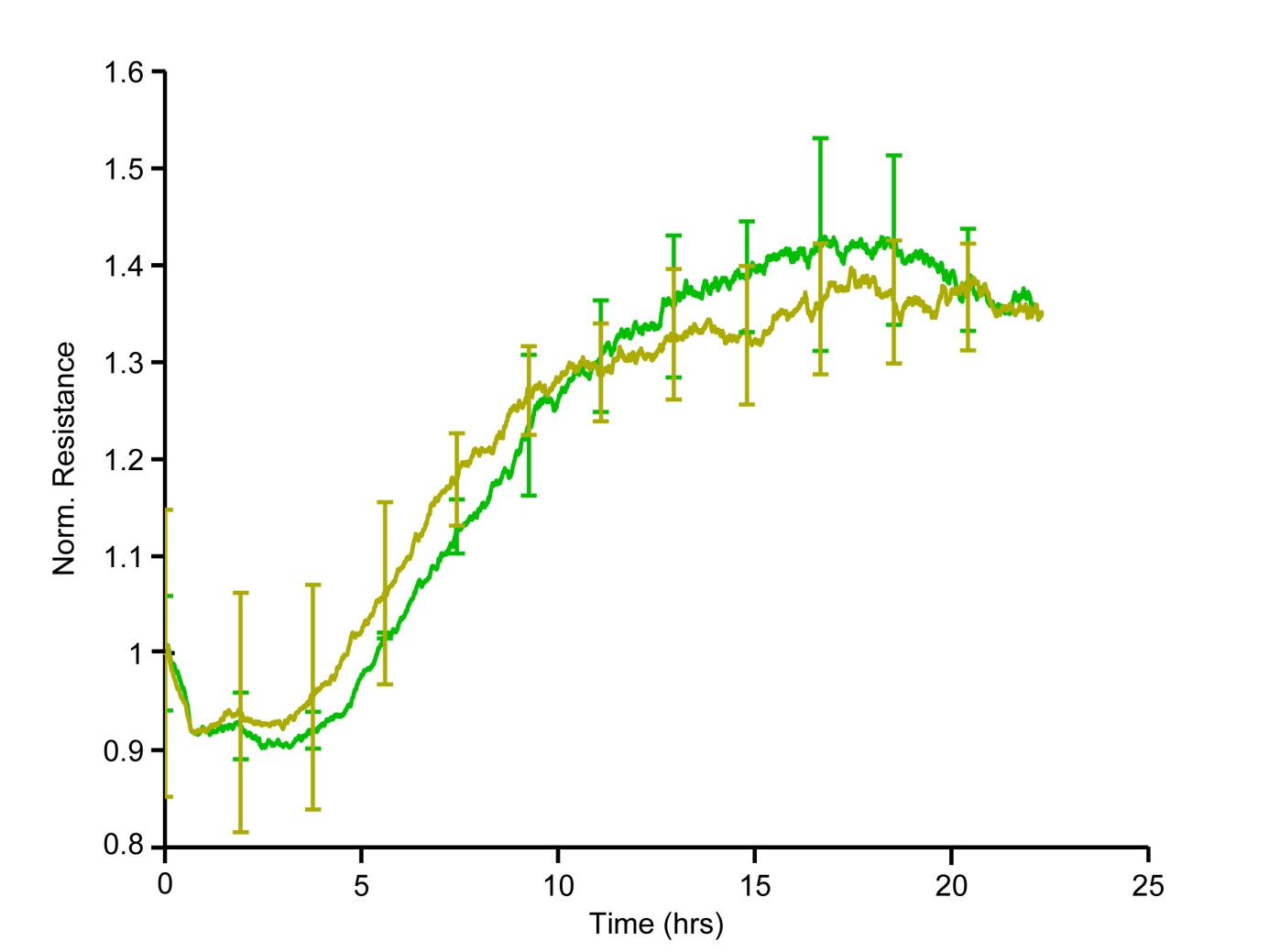


**S1 Fig. HCAEC monolayer formation in ECIS arrays.**

Supplement: S1 Fig — Immediately after seeding HCAEC into 8W10E+ arrays with a density of 80,000–90,000 cells/well, resistance measurements (in Ohms) were started and are shown as normalized resistance (subsequent values were divided by initial values). Increase in resistance over time indicates an increase in the formation of intercellular contacts. The steady state of resistance represents a tight monolayer stage exhibiting stable barrier function. Each single curve represents the resistance measurements conducted in duplicate wells which were grouped and averaged. Error bars of curves represent SD. Figures shown depict the resistance measurements conducted at 4000 Hz. Green and yellow, non-treated. (DOCX) [file pone.0156002.s001.docx]

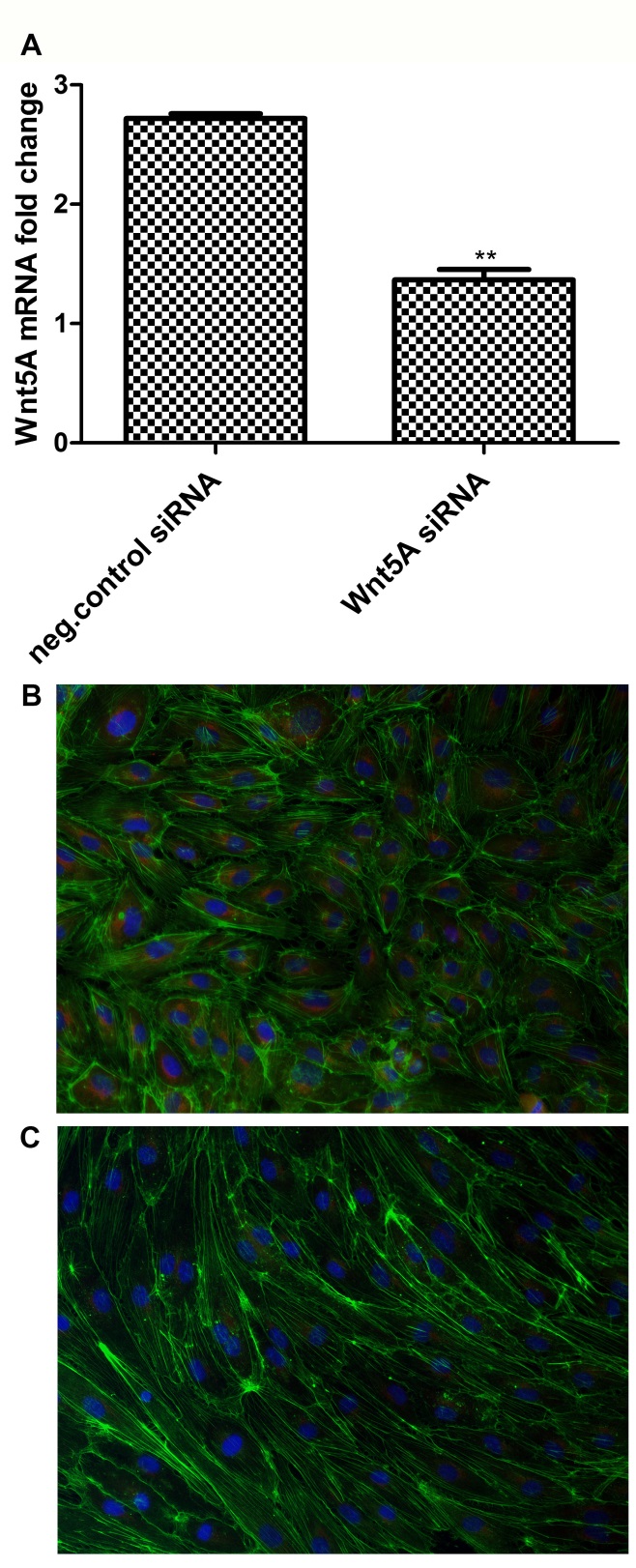


**S2 Fig.** **Wnt5A knockdown efficiency.**

Supplement: S2 Fig — (A) Expression levels of Wnt5A mRNA in HCAEC transfected with 5 nM negative (neg.) control siRNA and Wnt5A siRNA. Data were obtained from three independent qRT-PCR experiments run with duplicate samples and expressed as the mean ± SEM. *P<0.005. Representative immunofluorescence staining depicting Wnt5A protein expression (red) in negative control siRNA transfected (B) and Wnt5A siRNA transfected (C) HCAEC. Green: F-actin, Blue: nuclei. Zeiss Axioskope, Magnification 20×. (DOCX) [file pone.0156002.s002.docx]
