# Supplementary material for: IL-4 Causes Hyperpermeability of Vascular Endothelial Cells through Wnt5A Signaling
Source: PLoS One. 2016 May 23;11(5):e0156002. doi: 10.1371/journal.pone.0156002 (PMC4877093; doi:10.1371/journal.pone.0156002)
Supplement: S3 Fig — Fold changes in the expression of Wnt5A mRNA in 4 h IL-4 treated HCAEC. Data were obtained from three independent qRT-PCR experiments run with duplicate samples and expressed as the mean ± SEM. *P<0.05. (DOCX) [file pone.0156002.s003.docx]

S3 Fig. Expression of Wnt5A in HCAEC after 4 h stimulation with IL-4.
