# Supplementary material for: IL-4 Causes Hyperpermeability of Vascular Endothelial Cells through Wnt5A Signaling
Source: PLoS One. 2016 May 23;11(5):e0156002. doi: 10.1371/journal.pone.0156002 (PMC4877093; doi:10.1371/journal.pone.0156002)
Supplement: S1 Table — (DOCX) [file pone.0156002.s004.docx]

S1 Table. The top 100 genes upregulated by IL-4 in HCAEC.

| Gene symbol | Accession | Sequence description | Fold change |
| --- | --- | --- | --- |
| PMCH | NM_002674 | Homo sapiens pro-melanin-concentrating hormone | 41,795143 |
| HAS3 | NM_005329 | Homo sapiens hyaluronan synthase 3 | 40,64191 |
| CCL26 | NM_006072 | Homo sapiens chemokine (C-C motif) ligand 26 | 37,32348 |
| OTOGL | NM_173591 | Homo sapiens otogelin-like | 28,089672 |
| TMTC1 | NM_175861 | Homo sapiens transmembrane and tetratricopeptide repeat containing 1 | 26,668077 |
| CH25H | NM_003956 | Homo sapiens cholesterol 25-hydroxylase | 21,65999 |
| VCAM1 | NM_001078 | Homo sapiens vascular cell adhesion molecule 1 | 19,443674 |
| DKK2 | NM_014421 | Homo sapiens dickkopf homolog 2 | 12,351812 |
| SLC10A7 | NM_032128 | Homo sapiens solute carrier family 10 (sodium/bile acid cotransporter family), member 7 | 12,2331505 |
| MASP1 | NM_001031849 | Homo sapiens mannan-binding lectin serine peptidase 1 | 11,580042 |
| COL3A1 | NM_000090 | Homo sapiens collagen, type III, alpha 1 | 10,082533 |
| WNT5A | NM_003392 | Homo sapiens wingless-type MMTV integration site family, member 5A | 9,96113 |
| PKD1L1 | NM_138295 | Homo sapiens polycystic kidney disease 1 like 1 | 9,657538 |
| SOCS1 | NM_003745 | Homo sapiens suppressor of cytokine signaling 1 | 9,610145 |
| IGF1 | NM_000618 | Homo sapiens insulin-like growth factor 1 | 9,396036 |
| ERAP1 | NM_016442 | Homo sapiens endoplasmic reticulum aminopeptidase 1 | 9,268832 |
| PLCB4 | NM_182797 | Homo sapiens phospholipase C, beta 4 | 8,71052 |
| HS3ST1 | NM_005114 | Homo sapiens heparan sulfate (glucosamine) 3-O-sulfotransferase 1 | 8,354389 |
| FILIP1L | NM_182909 | Homo sapiens filamin A interacting protein 1-like | 8,258449 |
| NCF2 | NM_000433 | Homo sapiens neutrophil cytosolic factor 2 | 8,030248 |
| MAP3K8 | NM_005204 | Homo sapiens mitogen-activated protein kinase kinase kinase 8 | 7,8828645 |
| BMP2 | NM_001200 | Homo sapiens bone morphogenetic protein 2 | 7,6503243 |
| LIFR | NM_002310 | Homo sapiens leukemia inhibitory factor receptor alpha | 7,4233665 |
| BATF3 | NM_018664 | Homo sapiens basic leucine zipper transcription factor, ATF-like 3 | 7,255049 |
| FOXC1 | NM_001453 | Homo sapiens forkhead box C1 | 7,21699 |
| CISH | NM_145071 | Homo sapiens cytokine inducible SH2-containing protein | 7,0423675 |
| EGR1 | NM_001964 | Homo sapiens early growth response 1 | 7,0303206 |
| MMP7 | NM_002423 | Homo sapiens matrix metallopeptidase 7 | 6,91629 |
| AGXT2L1 | NM_031279 | Homo sapiens alanine-glyoxylate aminotransferase 2-like 1 | 6,9126115 |
| FAM115C | NM_173678 | Homo sapiens family with sequence similarity 115, member C | 6,896242 |
| SELP | NM_003005 | Homo sapiens selectin P | 6,8684745 |
| VSTM1 | NM_198481 | Homo sapiens V-set and transmembrane domain containing 1 | 6,8496885 |
| RTP4 | NM_022147 | Homo sapiens receptor (chemosensory) transporter protein 4 | 6,7024455 |
| CHRNB2 | NM_000748 | Homo sapiens cholinergic receptor, nicotinic, beta 2 | 6,6299343 |
| VIT | NM_001177972 | Homo sapiens vitrin | 6,553535 |
| GDF6 | NM_001001557 | Homo sapiens growth differentiation factor 6 | 6,516541 |
| SLC1A6 | NM_005071 | Homo sapiens solute carrier family 1 (high affinity aspartate/glutamate transporter), member 6 | 6,510828 |
| IGFL2 | NM_001002915 | Homo sapiens IGF-like family member 2 | 6,4454446 |
| EFCAB9 | NM_001171183 | Homo sapiens EF-hand calcium binding domain 9 | 6,444065 |
| OLFML2A | NM_182487 | Homo sapiens olfactomedin-like 2A | 6,3796315 |
| MYH11 | NM_001040114 | Homo sapiens myosin, heavy chain 11 | 6,3696566 |
| SP5 | NM_001003845 | Homo sapiens Sp5 transcription factor | 6,3607006 |
| C5orf46 | NM_206966 | Homo sapiens chromosome 5 open reading frame 46 | 6,228292 |
| UBD | NM_006398 | Homo sapiens ubiquitin D | 6,2165623 |
| C11orf70 | NM_001195005 | Homo sapiens chromosome 11 open reading frame 70 | 6,2152133 |
| TMTC1 | NM_175861 | Homo sapiens transmembrane and tetratricopeptide repeat containing 1 | 6,153664 |
| DACT1 | NM_016651 | Homo sapiens dapper, antagonist of beta-catenin, homolog 1 | 6,1239324 |
| MTMR7 | NM_004686 | Homo sapiens myotubularin related protein 7 | 6,1005383 |
| POU4F1 | NM_006237 | Homo sapiens POU class 4 homeobox 1 | 5,999347 |
| CXCR7 | NM_020311 | Homo sapiens chemokine (C-X-C motif) receptor 7 | 5,9171534 |
| PCDHGC4 | NM_032406 | Homo sapiens protocadherin gamma subfamily C, 4 | 5,8364773 |
| FABP4 | NM_001442 | Homo sapiens fatty acid binding protein 4 | 5,8192124 |
| CCL11 | NM_002986 | Homo sapiens chemokine (C-C motif) ligand 11 | 5,7279153 |
| IL6ST | NM_002184 | Homo sapiens interleukin 6 signal transducer | 5,718929 |
| DUOX1 | NM_017434 | Homo sapiens dual oxidase 1 | 5,7007294 |
| TSLP | NM_033035 | Homo sapiens thymic stromal lymphopoietin | 5,688645 |
| FXYD1 | NM_005031 | Homo sapiens FXYD domain containing ion transport regulator 1 | 5,568317 |
| KCND2 | NM_012281 | Homo sapiens potassium voltage-gated channel, Shal-related subfamily, member 2 | 5,4865217 |
| NAP1L3 | NM_004538 | Homo sapiens nucleosome assembly protein 1-like 3 | 5,437414 |
| SOX12 | NM_006943 | Homo sapiens SRY (sex determining region Y)-box 12 | 5,4160028 |
| TMEM87B | NM_032824 | Homo sapiens transmembrane protein 87B | 5,389064 |
| C22orf33 | NM_178552 | Homo sapiens chromosome 22 open reading frame 33 | 5,3850923 |
| ELMOD1 | NM_018712 | Homo sapiens ELMO/CED-12 domain containing 1 | 5,357257 |
| INHBA | NM_002192 | Homo sapiens inhibin, beta A | 5,324977 |
| HTR3C | NM_130770 | Homo sapiens 5-hydroxytryptamine (serotonin) receptor 3, family member C | 5,283623 |
| C2orf81 | NM_001145054 | Homo sapiens chromosome 2 open reading frame 81 | 5,1805587 |
| PTCH1 | NM_001083602 | Homo sapiens patched 1 | 5,1540847 |
| CCL20 | NM_004591 | Homo sapiens chemokine (C-C motif) ligand 20 | 5,0769067 |
| ANKRD55 | NM_024669 | Homo sapiens ankyrin repeat domain 55 | 4,962989 |
| C2CD4A | NM_207322 | Homo sapiens C2 calcium-dependent domain containing 4A | 4,910864 |
| HTR2B | NM_000867 | Homo sapiens 5-hydroxytryptamine (serotonin) receptor 2B | 4,856557 |
| IL6 | NM_000600 | Homo sapiens interleukin 6 | 4,831578 |
| C17orf109 | NM_001162995 | Homo sapiens chromosome 17 open reading frame 109 | 4,821182 |
| C6 | NM_000065 | Homo sapiens complement component 6 | 4,8188324 |
| MYOM1 | NM_003803 | Homo sapiens myomesin 1 | 4,792512 |
| LOX | NM_002317 | Homo sapiens lysyl oxidase | 4,7536435 |
| C1orf172 | NM_152365 | Homo sapiens chromosome 1 open reading frame 172 | 4,7347064 |
| RBPMS2 | NM_194272 | Homo sapiens RNA binding protein with multiple splicing 2 | 4,720724 |
| ZNF415 | NM_001136038 | Homo sapiens zinc finger protein 415 | 4,7036552 |
| IL1RL1 | NM_016232 | Homo sapiens interleukin 1 receptor-like 1 | 4,659585 |
| AMIGO2 | NM_181847 | Homo sapiens adhesion molecule with Ig-like domain 2 | 4,650363 |
| MYB | NM_005375 | Homo sapiens v-myb myeloblastosis viral oncogene homolog | 4,6442075 |
| ZFR2 | NM_015174 | Homo sapiens zinc finger RNA binding protein 2 | 4,6371136 |
| SERPINB13 | NM_012397 | Homo sapiens serpin peptidase inhibitor, clade B (ovalbumin), member 13 | 4,599599 |
| CRHBP | NM_001882 | Homo sapiens corticotropin releasing hormone binding protein | 4,5980325 |
| FSCN3 | NM_020369 | Homo sapiens fascin homolog 3, actin-bundling protein, testicular | 4,592466 |
| DBF4 | NM_006716 | Homo sapiens DBF4 homolog | 4,5902414 |
| LRP1B | NM_018557 | Homo sapiens low density lipoprotein receptor-related protein 1B | 4,5893173 |
| AIM1 | NM_001624 | Homo sapiens absent in melanoma 1 | 4,5863357 |
| KIR2DS4 | NM_012314 | Homo sapiens killer cell immunoglobulin-like receptor, two domains, short cytoplasmic tail, 4 | 4,5634003 |
| HIST2H2BE | NM_003528 | Homo sapiens histone cluster 2, H2be | 4,558278 |
| NUP62CL | NM_017681 | Homo sapiens nucleoporin 62kDa C-terminal like | 4,5011163 |
| BCL11A | NM_022893 | Homo sapiens B-cell CLL/lymphoma 11A | 4,495745 |
| FPR2 | NM_001462 | Homo sapiens formyl peptide receptor 2 | 4,471268 |
| EMR2 | NM_013447 | Homo sapiens egf-like module containing, mucin-like, hormone receptor-like 2 | 4,437741 |
| FLRT3 | NM_198391 | Homo sapiens fibronectin leucine rich transmembrane protein 3 | 4,4005938 |
| OXTR | NM_000916 | Homo sapiens oxytocin receptor | 4,391427 |
| ADAMTSL3 | NM_207517 | Homo sapiens ADAMTS-like 3 | 4,3507504 |
| SPDYE5 | NM_001099435 | Homo sapiens speedy homolog E5 | 4,3375187 |
| EFCAB3 | NM_173503 | Homo sapiens EF-hand calcium binding domain 3 | 4,3256946 |
