# Supplementary material for: IL-4 Causes Hyperpermeability of Vascular Endothelial Cells through Wnt5A Signaling
Source: PLoS One. 2016 May 23;11(5):e0156002. doi: 10.1371/journal.pone.0156002 (PMC4877093; doi:10.1371/journal.pone.0156002)
Supplement: S2 Table — (DOCX) [file pone.0156002.s005.docx]

S2 Table. The top 100 genes downregulated by IL-4 in HCAEC.

| Gene symbol | Accession | Sequence description | Fold change |
| --- | --- | --- | --- |
| LRRC14B | NM_001080478 | Homo sapiens leucine rich repeat containing 14B | -12,945567 |
| DNAH2 | NM_020877 | Homo sapiens dynein, axonemal, heavy chain 2 | -9,480801 |
| TMEM191B | NM_001242313 | Homo sapiens transmembrane protein 191B | -8,606891 |
| FAM47B | NM_152631 | Homo sapiens family with sequence similarity 47, member B | -8,436531 |
| GPR116 | NM_001098518 | Homo sapiens G protein-coupled receptor 116 | -7,9685755 |
| TTC9 | NM_015351 | Homo sapiens tetratricopeptide repeat domain 9 | -7,767549 |
| NOG | NM_005450 | Homo sapiens noggin | -7,578256 |
| OR13C8 | NM_001004483 | Homo sapiens olfactory receptor, family 13, subfamily C, member 8 | -7,5168667 |
| GPRC6A | NM_148963 | Homo sapiens G protein-coupled receptor, family C, group 6, member A | -7,467667 |
| CSN3 | NM_005212 | Homo sapiens casein kappa | -7,366031 |
| TMEM236 | NM_001098844 | Homo sapiens transmembrane protein 236 | -7,1928277 |
| PRKG2 | NM_006259 | Homo sapiens protein kinase, cGMP-dependent, type II | -7,114787 |
| DGKB | NM_145695 | Homo sapiens diacylglycerol kinase, beta 90kDa | -7,055235 |
| CSTL1 | NM_138283 | Homo sapiens cystatin-like 1 | -7,038949 |
| GALNT14 | NM_024572 | Homo sapiens UDP-N-acetyl-alpha-D-galactosamine:polypeptide N-acetylgalactosaminyltransferase 14 | -6,925887 |
| N4BP3 | NM_015111 | Homo sapiens NEDD4 binding protein 3 | -6,8579197 |
| ARHGEF38 | NM_001242729 | Homo sapiens Rho guanine nucleotide exchange factor (GEF) 38 | -6,75289 |
| MRVI1 | NM_130385 | Homo sapiens murine retrovirus integration site 1 homolog | -6,6988463 |
| EPN3 | NM_017957 | Homo sapiens epsin 3 | -6,594775 |
| FSIP2 | NM_173651 | Homo sapiens fibrous sheath interacting protein 2 | -6,4968653 |
| SGPP2 | NM_152386 | Homo sapiens sphingosine-1-phosphate phosphatase 2 | -5,6272907 |
| STARD6 | NM_139171 | Homo sapiens StAR-related lipid transfer (START) domain containing 6 | -5,626096 |
| SLC22A12 | NM_144585 | Homo sapiens solute carrier family 22 (organic anion/urate transporter), member 12 | -5,625856 |
| FCRL6 | NM_001004310 | Homo sapiens Fc receptor-like 6 | -5,603581 |
| GPR151 | NM_194251 | Homo sapiens G protein-coupled receptor 151 | -5,515336 |
| TTC16 | NM_144965 | Homo sapiens tetratricopeptide repeat domain 16 | -5,5019436 |
| SGCD | NM_000337 | Homo sapiens sarcoglycan, delta | -5,411149 |
| OSTN | NM_198184 | Homo sapiens osteocrin | -5,3031754 |
| PCDHGB1 | NM_032095 | Homo sapiens protocadherin gamma subfamily B, 1 | -5,2786555 |
| NEK10 | NM_199347 | Homo sapiens NIMA (never in mitosis gene a)- related kinase 10 | -5,2474537 |
| FREM3 | NM_001168235 | Homo sapiens FRAS1 related extracellular matrix 3 | -5,1939254 |
| NLRP10 | NM_176821 | Homo sapiens NLR family, pyrin domain containing 10 | -5,183514 |
| REM1 | NM_014012 | Homo sapiens RAS (RAD and GEM)-like GTP-binding 1 | -5,148359 |
| TCHHL1 | NM_001008536 | Homo sapiens trichohyalin-like 1 | -5,1217394 |
| ZNF846 | NM_001077624 | Homo sapiens zinc finger protein 846 | -5,025068 |
| LEKR1 | NM_001004316 | Homo sapiens leucine, glutamate and lysine rich 1 | -5,012441 |
| TPTE2 | NM_199254 | Homo sapiens transmembrane phosphoinositide 3-phosphatase and tensin homolog 2 | -5,006573 |
| TAS2R5 | NM_018980 | Homo sapiens taste receptor, type 2, member 5 | -5,004593 |
| CEACAM3 | NM_001815 | Homo sapiens carcinoembryonic antigen-related cell adhesion molecule 3 | -4,9621882 |
| ZNF551 | NM_138347 | Homo sapiens zinc finger protein 551 | -4,959369 |
| SEMA4D | NM_006378 | Homo sapiens sema domain, immunoglobulin domain (Ig), transmembrane domain (TM) and short cytoplasmic domain, (semaphorin) 4D | -4,948316 |
| KIT | NM_000222 | Homo sapiens v-kit Hardy-Zuckerman 4 feline sarcoma viral oncogene homolog | -4,931853 |
| ANKRD20A2 | NM_001012421 | Homo sapiens ankyrin repeat domain 20 family, member A2 | -4,89075 |
| EPHA10 | NM_173641 | Homo sapiens EPH receptor A10 | -4,870359 |
| SEMA5B | NM_001031702 | Homo sapiens sema domain, seven thrombospondin repeats (type 1 and type 1-like), transmembrane domain (TM) and short cytoplasmic domain, (semaphorin) 5B | -4,8688235 |
| SPRR4 | NM_173080 | Homo sapiens small proline-rich protein 4 | -4,8533287 |
| MYL3 | NM_000258 | Homo sapiens myosin, light chain 3 | -4,8104806 |
| PLB1 | NM_153021 | Homo sapiens phospholipase B1 | -4,73602 |
| CHI3L1 | NM_001276 | Homo sapiens chitinase 3-like 1 | -4,7334285 |
| FMN2 | NM_020066 | Homo sapiens formin 2 | -4,7102337 |
| INMT | NM_001199219 | Homo sapiens indolethylamine N-methyltransferase | -4,67023 |
| ZCCHC13 | NM_203303 | Homo sapiens zinc finger, CCHC domain containing 13 | -4,646102 |
| HAPLN1 | NM_001884 | Homo sapiens hyaluronan and proteoglycan link protein 1 | -4,6067057 |
| DNAJB8 | NM_153330 | Homo sapiens DnaJ (Hsp40) homolog, subfamily B, member 8 | -4,6007133 |
| RGS7BP | NM_001029875 | Homo sapiens regulator of G-protein signaling 7 binding protein | -4,581259 |
| XAGE5 | NM_130775 | Homo sapiens X antigen family, member 5 | -4,4819264 |
| RNASE2 | NM_002934 | Homo sapiens ribonuclease, RNase A family, 2 | -4,4812913 |
| MUC12 | NM_001164462 | Homo sapiens mucin 12, cell surface associated | -4,476315 |
| ALPK2 | NM_052947 | Homo sapiens alpha-kinase 2 | -4,464628 |
| FMR1NB | NM_152578 | Homo sapiens fragile X mental retardation 1 neighbor | -4,4601526 |
| ACOT6 | NM_001037162 | Homo sapiens acyl-CoA thioesterase 6 | -4,4558067 |
| ZNF436 | NM_001077195 | Homo sapiens zinc finger protein 436 | -4,439032 |
| PPP1R1A | NM_006741 | Homo sapiens protein phosphatase 1, regulatory (inhibitor) subunit 1A | -4,4109473 |
| HS6ST3 | NM_153456 | Homo sapiens heparan sulfate 6-O-sulfotransferase 3 | -4,379276 |
| CXorf22 | NM_152632 | Homo sapiens chromosome X open reading frame 22 | -4,3745127 |
| KAZN | NM_015209 | Homo sapiens kazrin, periplakin interacting protein | -4,3690753 |
| SPANXB2 | NM_145664 | Homo sapiens SPANX family, member B2 | -4,362014 |
| SPINK5 | NM_001127698 | Homo sapiens serine peptidase inhibitor, Kazal type 5 | -4,3541923 |
| DDIT4L | NM_145244 | Homo sapiens DNA-damage-inducible transcript 4-like | -4,311884 |
| VCX2 | NM_016378 | Homo sapiens variable charge, X-linked 2 | -4,2942934 |
| CCKBR | NM_176875 | Homo sapiens cholecystokinin B receptor | -4,259044 |
| KIAA1199 | NM_018689 | Homo sapiens KIAA1199 | -4,2481823 |
| ATP2A3 | NM_174958 | Homo sapiens ATPase, Ca++ transporting, ubiquitous | -4,239258 |
| SPANXN3 | NM_001009609 | Homo sapiens SPANX family, member N3 | -4,2082605 |
| KRT6B | NM_005555 | Homo sapiens keratin 6B | -4,2064652 |
| APLN | NM_017413 | Homo sapiens apelin | -4,198813 |
| PCDHA1 | NM_031410 | Homo sapiens protocadherin alpha 1 | -4,17986 |
| ZP2 | NM_003460 | Homo sapiens zona pellucida glycoprotein 2 | -4,1756864 |
| SMOC2 | NM_022138 | Homo sapiens SPARC related modular calcium binding 2 | -4,1644816 |
| SPINK4 | NM_014471 | Homo sapiens serine peptidase inhibitor, Kazal type 4 | -4,1478534 |
| C1orf49 | NM_032126 | Homo sapiens chromosome 1 open reading frame 49 | -4,146102 |
| CYP3A43 | NM_022820 | Homo sapiens cytochrome P450, family 3, subfamily A, polypeptide 43 | -4,1281586 |
| GPR112 | NM_153834 | Homo sapiens G protein-coupled receptor 112 | -4,1099563 |
| ZNF253 | NM_021047 | Homo sapiens zinc finger protein 253 | -4,0930114 |
| TPD52L3 | NM_033516 | Homo sapiens tumor protein D52-like 3 | -4,05051 |
| GZMK | NM_002104 | Homo sapiens granzyme K | -4,0004787 |
| LOC55908 | NM_018687 | Homo sapiens hepatocellular carcinoma-associated gene TD26 | -3,9874408 |
| SPEF2 | NM_024867 | Homo sapiens sperm flagellar 2 | -3,9677868 |
| ZNF208 | NM_007153 | Homo sapiens zinc finger protein 208 | -3,9518707 |
| PTCHD2 | NM_020780 | Homo sapiens patched domain containing 2 | -3,9511278 |
| PARVG | NM_022141 | Homo sapiens parvin, gamma (PARVG), transcript variant 1, mRNA | -3,9182448 |
| FTCD | NM_206965 | Homo sapiens formiminotransferase cyclodeaminase (FTCD), transcript variant A, mRNA | -3,9056072 |
| NKAIN2 | NM_001040214 | Homo sapiens Na+/K+ transporting ATPase interacting 2 (NKAIN2), transcript variant 1, mRNA | -3,877624 |
| DMGDH | NM_013391 | Homo sapiens dimethylglycine dehydrogenase (DMGDH), nuclear gene encoding mitochondrial protein, mRNA | -3,8753538 |
| SPATA13 | NM_001166271 | Homo sapiens spermatogenesis associated 13 (SPATA13), transcript variant 1, mRNA | -3,860474 |
| PTPN3 | NM_002829 | Homo sapiens protein tyrosine phosphatase, non-receptor type 3 (PTPN3), transcript variant 1, mRNA | -3,79502 |
| C10orf99 | NM_207373 | Homo sapiens chromosome 10 open reading frame 99 (C10orf99), mRNA | -3,792719 |
| MYH4 | NM_017533 | Homo sapiens myosin, heavy chain 4, skeletal muscle (MYH4), mRNA | -3,7923658 |
| C5orf52 | NM_001145132 | Homo sapiens chromosome 5 open reading frame 52 (C5orf52), mRNA | -3,7702193 |
| HAVCR1 | NM_012206 | Homo sapiens hepatitis A virus cellular receptor 1 (HAVCR1), transcript variant 1, mRNA | -3,7554414 |
