# Supplementary material for: IL-4 Causes Hyperpermeability of Vascular Endothelial Cells through Wnt5A Signaling
Source: PLoS One. 2016 May 23;11(5):e0156002. doi: 10.1371/journal.pone.0156002 (PMC4877093; doi:10.1371/journal.pone.0156002)
Supplement: S3 Table — (DOCX) [file pone.0156002.s006.docx]

S3 Table. Regulation of genes coding for transcription factors in IL-4 treated HCAEC.

| Gene symbol | Accession | Sequence description | Fold change |
| --- | --- | --- | --- |
| EGR1 | NM_001964 | Homo sapiens early growth response 1 | 7.030321 |
| FOSB | NM_006732 | Homo sapiens FBJ murine osteosarcoma viral oncogene homolog B | 3.307321 |
| GATA3 | NM_001002295 | Homo sapiens GATA binding protein 3 | 3.068881 |
